# Genetic diversity and population structure of advanced clones selected over forty years by a potato breeding program in the USA

**DOI:** 10.1038/s41598-021-87284-x

**Published:** 2021-04-16

**Authors:** Jeewan Pandey, Douglas C. Scheuring, Jeffrey W. Koym, Joseph Coombs, Richard G. Novy, Asunta L. Thompson, David G. Holm, David S. Douches, J. Creighton Miller, M. Isabel Vales

**Affiliations:** 1 Department of Horticultural Sciences, Texas A&M University, College Station, TX 77843-2133 USA; 2 Texas A&M AgriLife Research and Extension Center, Lubbock, TX 79403 USA; 3 Department of Plant, Soil, and Microbial Sciences, Michigan State University, East Lansing, MI 48824 USA; 4 USDA-Agricultural Research Service, Small Grains and Potato Germplasm Research, Aberdeen, ID 83210 USA; 5 Department of Plant Sciences, North Dakota State University, Fargo, ND 58108 USA; 6 San Luis Valley Research Center, Department of Horticulture and Landscape Architecture, Colorado State University, Center, CO 81125 USA

**Keywords:** Molecular biology, Plant sciences

## Abstract

Knowledge regarding genetic diversity and population structure of breeding materials is essential for crop improvement. The Texas A&M University Potato Breeding Program has a collection of advanced clones selected and maintained in-vitro over a 40-year period. Little is known about its genetic makeup and usefulness for the current breeding program. In this study, 214 potato clones were genotyped with the Infinium Illumina 22 K V3 Potato Array. After filtering, a total of 10,106 single nucleotide polymorphic (SNP) markers were used for analysis. Heterozygosity varied by SNP, with an overall average of 0.59. Three groups of tetraploid clones primarily based on potato market classes, were detected using STRUCTURE software and confirmed by discriminant analysis of principal components. The highest coefficient of differentiation observed between the groups was 0.14. Signatures of selection were uncovered in genes controlling potato flesh and skin color, length of plant cycle and tuberization, and carbohydrate metabolism. A core set of 43 clones was obtained using Core Hunter 3 to develop a sub-collection that retains similar genetic diversity as the whole population, minimize redundancies, and facilitates long-term conservation of genetic resources. The comprehensive molecular characterization of our breeding clone bank collection contributes to understanding the genetic diversity of existing potato resources. This analysis could be applied to other breeding programs and assist in the selection of parents, fingerprinting, protection, and management of the breeding collections.

## Introduction

Potato (*Solanum tuberosum* L.) is the world’s fourth most important crop after maize, rice, and wheat^1^. Worldwide, over one billion people consume potatoes as a staple food^2^. Potatoes, the leading vegetable crop in the United States, are grown commercially in 30 states. Idaho grows more potatoes than any other state, followed by Washington, North Dakota, Wisconsin, and Colorado^2^. Even though potato production in Texas is comparatively lower (approximately 8,000 ha), growers can harvest and provide fresh potatoes to the market earlier in the growing season than other states, and often receive two to three times higher prices than growers from Northern States^2^.

The Texas A&M University (TAMU) Potato Breeding Program was established by the late J. Creighton Miller Jr., Ph.D. in 1972 aiming to provide improved early maturing cultivars with high yield and quality that would enable Texas potato producers to remain viable and competitive, and to supply superior products to consumers. In recent years, the emphasis has been placed on increasing yield and quality in addition to disease/pest resistances like Potato Virus Y (PVY), nematodes (*Globodera rostochiensis* and *Globodera pallida*), late blight (*Phytophthora infestans*), potato psyllid (*Bactericera cockerelli* carrying *Candidatus* Liberibacter, the causal agent of the zebra chip disease), high-temperature tolerance, cold sweetening resistance, health and nutritional properties, and broad adaptability. The TAMU Potato Breeding program has developed/co-developed and released 17 cultivars, including clonal selections. Some of them make up a substantial and increasing share of the regional/national potato production and have become important contributors to the economies of several states. Of all the cultivars released over the past 15 years by the 12 US potato breeding programs, those developed by the Texas program have ranked in the top four to five nationally in the total area approved for seed certification over the past several years^3^. This has been due, in large measure, to the popularity of the four Texas Russet Norkotah strains (Russet Norkotah 112, Russet Norkotah 223, Russet Norkotah 278, and Russet Norkotah 296) with improved plant type to withstand environmental stresses. The Texas Russet Norkotah strains with increased vine vigor and some resistance to early dying (*Verticillium* wilt) are an outstanding early market alternative to the standard Russet Norkotah variety^4^.

Despite many available potato cultivars, there is a need for new cultivars. New cultivars must produce high yields under low inputs, have disease and pest resistance, and environmental stress tolerance such as high or low temperature, drought, and salinity. If possible, they should also have improved nutritional and health properties^5^. Exploration of potato genetic diversity has been proposed to create new varieties well adapted to these challenges, and also to better manage these collections. The development of new, improved varieties is done through breeding, which involves identifying superior and complementary parents from the available germplasm and crossing them to generate variability and permit selection of clones combining trait of interest. Breeders maintain valuable germplasm in tissue culture for long-term conservation of genetic resources, and also to initiate limited generation seed production of potato varieties from disease-free stocks. Thus, breeders have to think strategically to capture allelic diversity from a smaller set of parent combinations. For this, a breeder can use genetic distance based on molecular markers to complement co-ancestry/pedigree analysis to avoid crossing closely related parents and hence prevent inbreeding depression and to ensure genetic variation for continued selection progress. Genetic distance-based criteria have also been strongly recommended for evaluation and creation of core sets^6^.

Further, the genetic characterization of clone bank collections is essential to assess their diversity and population structure. The identification of suitable genotypes from the study could serve as a source of new alleles in potato breeding programs. Molecular markers have been used to test the genetic diversity of potatoes. Recent advances in the development of high‐throughput genotyping platforms together with whole-genome coverage and affordability have turned single nucleotide polymorphisms (SNPs) into one of the most promising tools for the investigation of genetic diversity. Several studies have implemented the Infinium Potato Array (Illumina Inc., San Diego, CA, USA) for genetic diversity studies. The 8 K SNP array distinguished diverse North American varieties based mainly on market classes^7^. Kolech et al.^8^ used the same set of 8 K SNPs to evaluate the genetic diversity of Ethiopian potato cultivars. Vos et al.^9^ developed a 20 K SNP array and used it to genotype a total of 569 potato genotypes and found fingerprints of the breeding history in recent breeding materials such as identification of introgression segments, selection, and founder signatures. Genetic diversity in the Colombian Central Collection of *Solanum tuberosum* L. using SNP markers found that the Andigena (autotetraploid) population was more genetically diverse, but less genetically sub structured than the Phureja (diploid) population^10^. Ellis et al.^11^ used the 12 K SNP array for fingerprinting and diversity analysis of the cultivated potato collection from the International Potato Center (CIP) in Peru and reported some genetic redundancies among individual accessions with some putative misclassified accessions. Recently, Igarashi et al.^12^ used the 12 K SNP array to characterize and compare 164 Japanese potatoes, including 70 breeding clones for chip processing with North American and European potatoes. Thus, the success of potato breeding depends on the understanding and use of the available gene pool of varieties and breeding clones. The Potato SNP array has been very useful for performing a robust and direct comparison of genetic diversity among different gene pools but has never been applied to the advanced clones selected over multiple years.

Further, with the availability of high-density genotype data, it is possible to identify regions of the genome that provide evidence of selective pressure commonly known as signatures of selection^13^. Different statistical approaches have been developed to identify selection footprints. According to Vitti et al.^14^, they are of three main types: (a) measures based on the allelic frequencies (e.g., Tajima’s D, PCAdapt), (b) measures based on the differentiation between and within species/groups (e.g., XP-EHH, Fst), and (c) within population/groups measures based on extended haplotype homozygosity (e.g., iHS). These methods have been applied to several crops, including wheat^15^, oat^16^, maize^17^, rice^18^, tomato^19^, and potato^20^. The PCAdapt method tests how much each variant is associated with population structure, assuming that outlier variants are indicative of local adaptation. It does not need grouping of individuals into populations and can handle admixed individuals^21^. The iHS approach measures the amount of extended haplotype homozygosity (EHH) for a given SNP within-population whereas XP-EHH compares the extended haplotype homozygosity between two populations^22^. Recent selection events in which haplotypes have almost or fully risen to fixation are detected by iHS and XP-EHH statistics^22^. Thus, methods for detecting evidence of selection provides a mechanism for highlighting genomic regions which are often associated with functional traits.

The goal of this research was to investigate potato varieties and advanced clones of the TAMU Potato Breeding Program (entered in the clone bank over 40 years of breeding) at the molecular level to assess genetic diversity for further genetic enhancement of important economic traits. In this study 214 TAMU potato clones were genotyped using 22 K SNP markers to (a) examine the genetic diversity and the population structure in the TAMU Potato Breeding clone bank collection, (b) to identify candidate loci under selection, (c) identify a “core set” to better manage the clone bank collection, and (d) check the accuracy of pedigree records of the clones.

## Results

Two hundred fourteen clones, including commercial and reference varieties maintained by the TAMU Potato Breeding Program, were genotyped with the Infinium 22 K V3 Potato Array. Stringent screening of the SNP markers using MAF in removed 10,669 SNP (50.7%) markers and additional filtering for more than 10% “No call rate” removed 252 (1.2%) SNP markers. After filtering, a total of 10,106 polymorphic SNP markers were selected for analysis (Supplementary Information Table S2).

### Genome-wide distribution of SNPs

**Figure 1:**
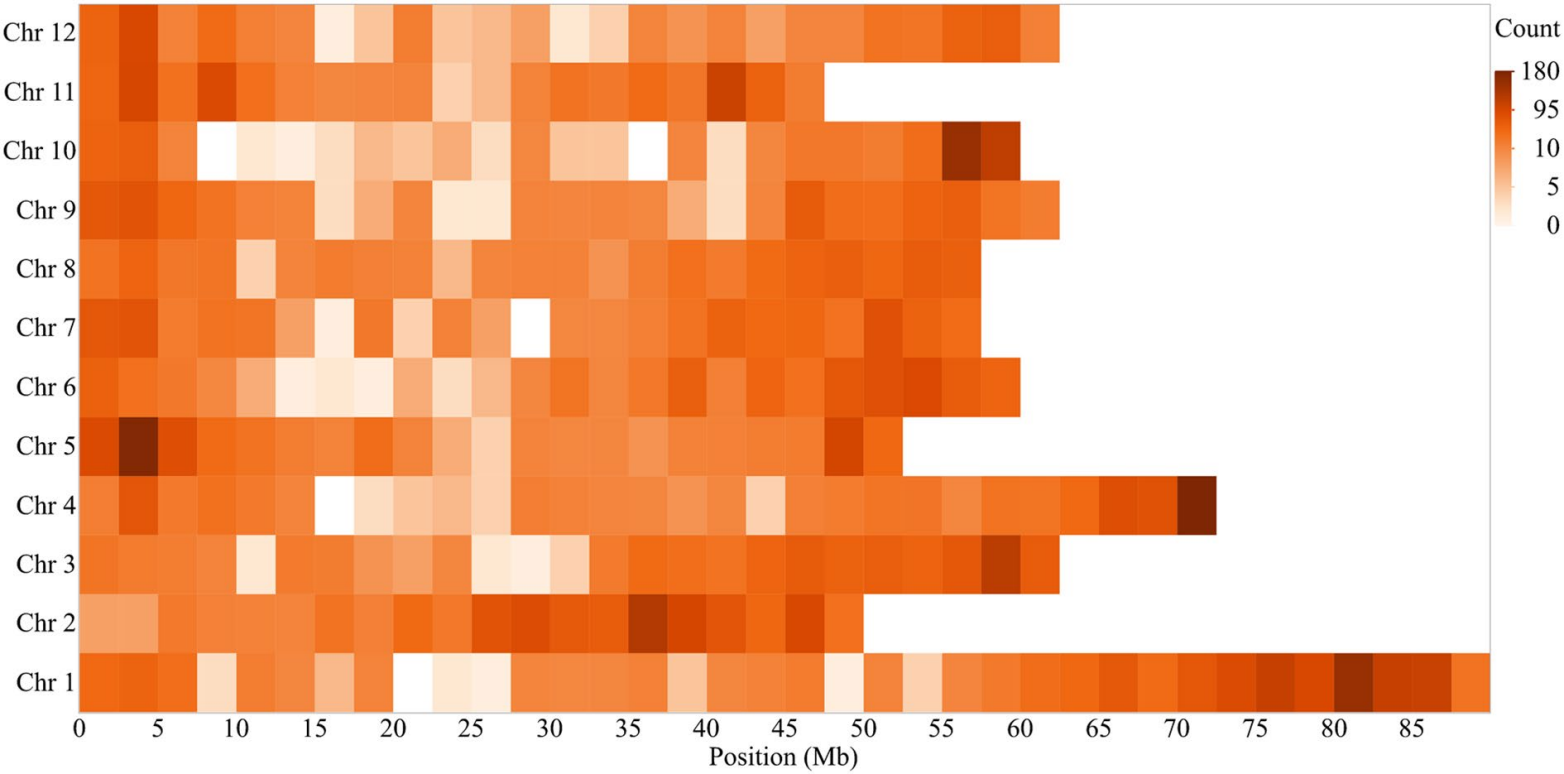
Heatmap of 10,106 SNPs across the twelve potato chromosomes. The color intensity indicates the density of markers in that segment of the chromosome (white, low density; maroon, high density). SNP density is shown to increase toward the ends of the chromosomes where gene density is higher.

The SNPs were distributed across the 12 chromosomes. 10,106 SNP markers (after filtering) were mapped to 12 chromosomes represented as the 12 pseudomolecules of the potato genome DMv4.03^23^ (Supplementary Information Table S3). Each chromosome had an average of 842 markers ranging from 1,389 markers on Chr. 1 to 617 on Chr. 10. The average distance between SNPs was 71 kb, but the SNP to SNP distribution was skewed: 39% of the marker to marker distances were less than 1 kb, and 18% were less than 10 kb. SNPs were enriched toward chromosome ends (Fig. 1).

### Evaluation of SNP characteristics

**Figure 2:**
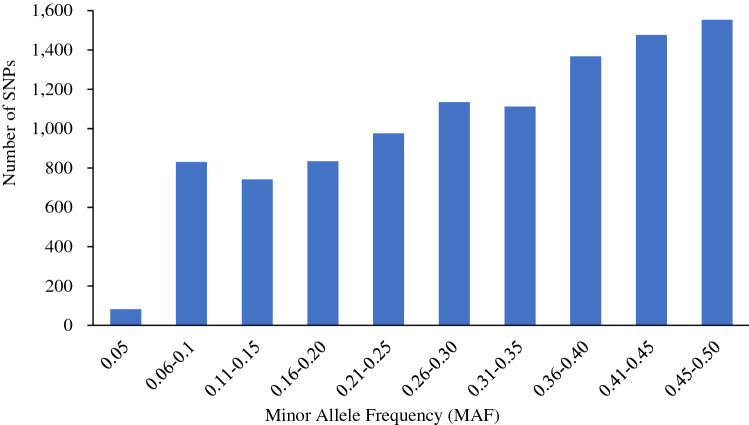
Distribution of minor allele frequency (MAF) of 10,106 SNPs in 214 tetraploid clones.

**Figure 3:**
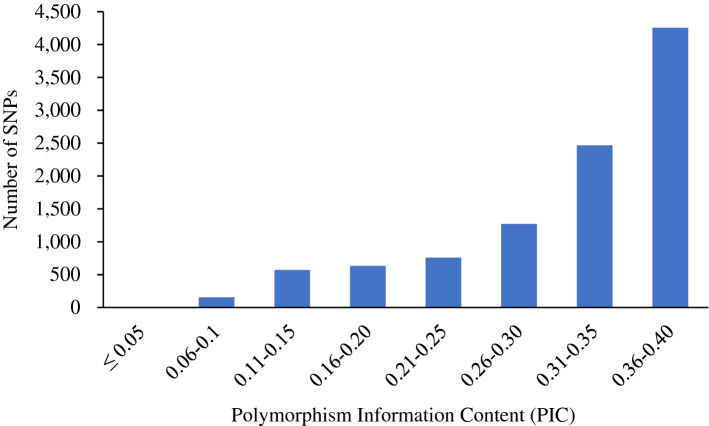
Distribution of polymorphic information content (PIC) values of 10,106 SNPs calculated for 214 tetraploid clones across 10,106 SNPs.

The mean expected heterozygosity value of the SNP markers was 0.39, ranging from 0.10 to 0.50. Minor allele frequency (MAF) ranged from 0.05 to 0.50, with a mean of 0.31 (Fig. 2; Supplementary Information Table S3). The polymorphic information content (PIC), which denotes the relative informativeness of each marker, ranged from 0.09 to 0.38 with a mean of 0.31 (Fig. 3; Supplementary Information Table S3). Most of the clones had high levels of heterozygosity, ranging from 0.22 to 0.80 with a mean of 0.59 (Supplementary Information Table S4). The mean heterozygosity values for different market classes were 0.62 (Chipping), 0.59 (Russet), and 0.58 (Red/Specialties). A clone (ATX91322-2Y/Y) with very low frequencies of simplex and triplex was found. Those two allelic classes are absent in diploids. ATX91322-2Y/Y produces very small potatoes, yellow skin, and very intense yellow flesh. Thus, we are declaring this clone as a diploid potato. The inbreeding coefficient was negative for many highly heterozygous clones ranging from − 1.00 to 0.44, with a mean of − 0.51 (Supplementary Information Table S4).

### Genetic diversity

Based on the diploid genotypic calls analysis using TASSEL, the average pairwise divergence among genotypes (π), at SNP locations, was 0.39. This represents the nucleotide diversity per assayed SNP in the clones. The expected number of polymorphic sites per nucleotide (θ), which estimates the mutation rate in the collection, was 0.169 with 10,106 segregating sites. Tajima’s D, which estimates the normalized measure of the difference between the observed (π) and expected (θ) nucleotide diversity was 4.29.

### Population structure analysis

**Figure 4:**
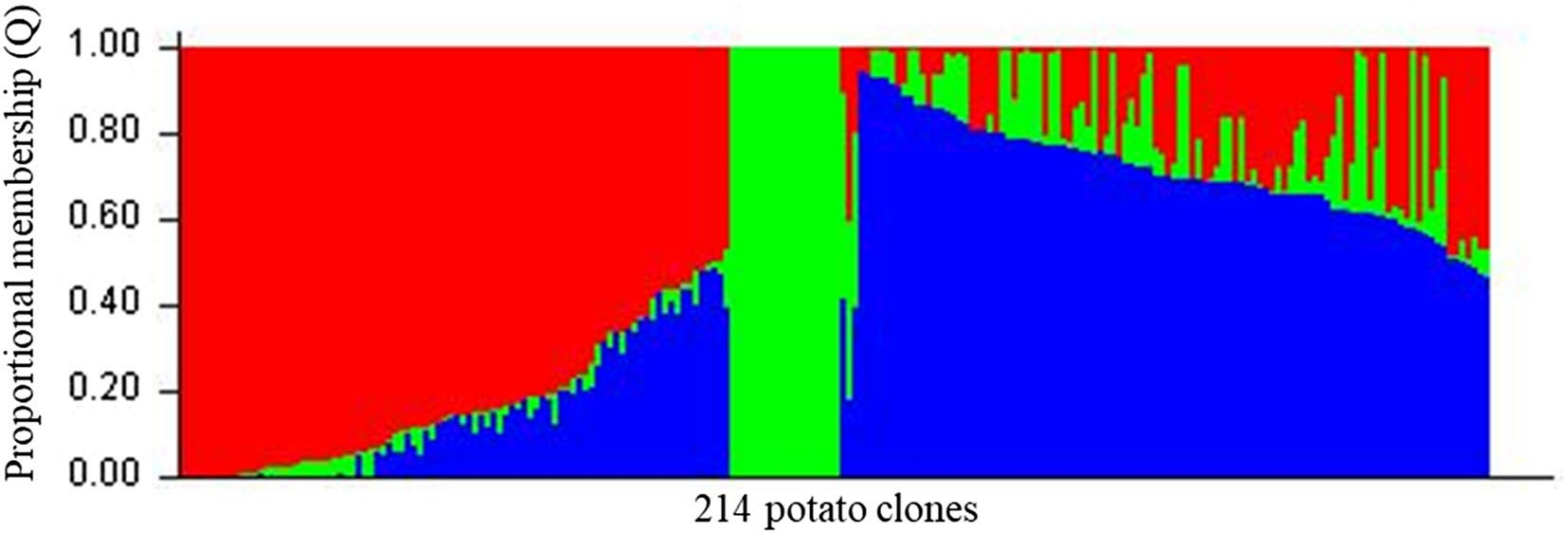
Proportional membership (Q) of each clone in the genetic clusters inferred by STRUCTURE (K = 3). This figure represents each individual as a vertical bar and its membership probability in each subpopulation. Individuals with the highest proportion of membership to a subpopulation 1 (red color) corresponded to clones with red, purple, and yellow skin; Individuals belonging mainly to subpopulation 2 (green) include Russet Norkotah strain selections, and; Individuals with predominate membership to subpopulation 3 (blue) were russet and chipping clones.

STRUCTURE analysis showed that the number of subpopulations (K) ranged from zero to ten when using the diploid genotyping model (AA, AB, BB; 10,106 SNP markers). The K value with the maximum likelihood was K = 3 (Fig. 4, Supplementary Fig. S1). Clones were assigned to a subpopulation if they had at least 50% membership within that group. Most of the reds, purples, and yellows (46.6% of total clones) grouped in subpopulation 1 (Red) (Fig. 4, Supplementary Information Table S5). For instance, the red skinned yellow flesh clone Sierra Rose, the purple skinned yellow flesh clone ATTX88654-2P/Y, and the yellow skin yellow flesh clone ATX91322-2Y/Y had complete membership in subpopulation 1. Russet Norkotah and its strain selections (8.4% of total clones) were grouped in a separate subpopulation 2 (Green) (Fig. 4, Supplementary Information Table S5). The majority of russet and chipping clones (40.6% of total clones) were grouped to subpopulation 3 (Blue) (Fig. 4; Supplementary Information Table S5). For instance, the chipping clone Atlantic and the russet clone Reveille Russet had complete membership to subpopulation 3. STRUCTURE analysis revealed significant admixtures in 4.67% of the total clones. e.g., White LaSoda, TX11454-9Ru/Y, and COTX87601-2Ru (Fig. 4; Supplementary Fig. S2; Supplementary Information Table S5).

### Discriminant analysis of principal components (DAPC) analysis

**Figure 5:**
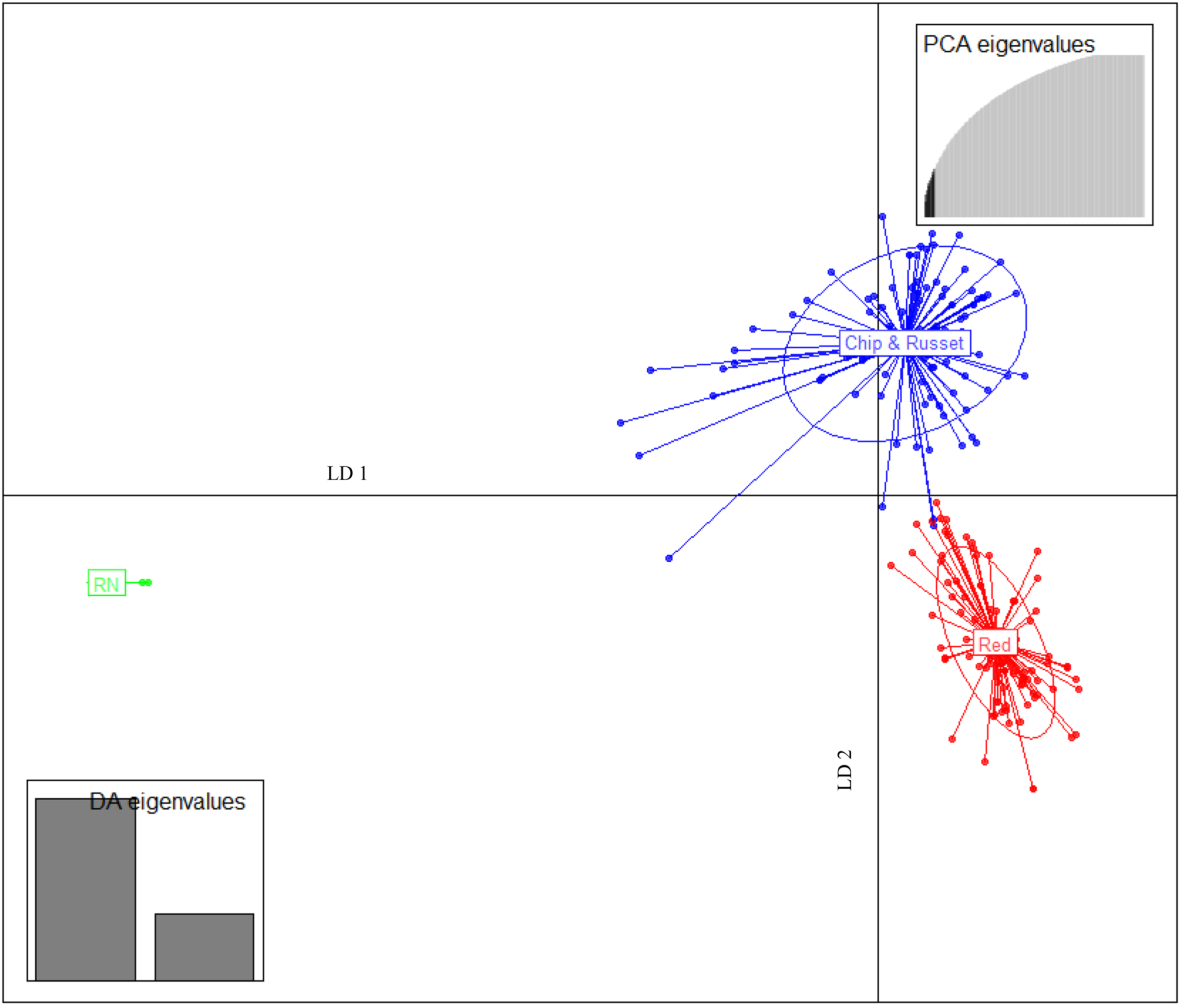
Discriminant analysis of principal components (DAPC) for 214 clones using *adegenet* R package^67^. The axes represent the first two linear discriminants (LD). Circumferences surround each group, and small solid dots represent individual clones. Labels inside circles indicate the different subpopulations identified by DAPC analysis (Chip & Russet = chipping and russet clones, Red = red/specialties, and RN = Russet Norkotah and its strains).

The lowest Bayesian information criterion (BIC) value obtained using *find.clusters* function was three (Supplementary Fig. S3), which was in concordance with the delta K obtained in STRUCTURE. These three clusters were used to analyze the DAPC (Fig. 5). Twenty principal components capturing 34.3% variance and two discriminant eigenvalues were retained. These values were confirmed by a cross-validation analysis (Supplementary Fig. S4). Genotypes had membership coefficients to each group ranging from 0.5 to 1, thus confirming low admixture and structured population (Supplementary Information Table S6). Exceptions to these values were clone NDTX059775-1 W (chipper with white flesh), COTX10118-4Wpe/Y (specialty with white skin purple eyes, and yellow flesh), COTX03079-1 W (chipper with white flesh), and COTX94216-1R (red skin white flesh) whose values were 0.36, 0.40, 0.43 and 0.44 respectively. In Fig. 5, Linear Discriminant 1 (LD 1) separated Russet Norkotah and Red groups from Chip & Russet group and Linear Discriminant 2 (LD 2) separated Red and Chip & Russet groups from the Russet Norkotah group. *SNPZIP* analysis detected 18 SNPs with the largest contribution to cluster identification. Two of them corresponded to LD 1, and the remaining 16 to the LD 2. Most of them annotated with known gene functions (Supplementary Information Table S7). The coefficient of genetic differentiation among groups was highest (0.14) between Red/Specialties and Russet Norkotah/strains, followed by Chip & Russet and Russet Norkotah/strains (0.10). The lowest value (0.02) was found between the Chip & Russet and Red/Specialties groups suggesting low genetic differentiation among them.

### Phylogenetic cluster analysis

**Figure 6:**
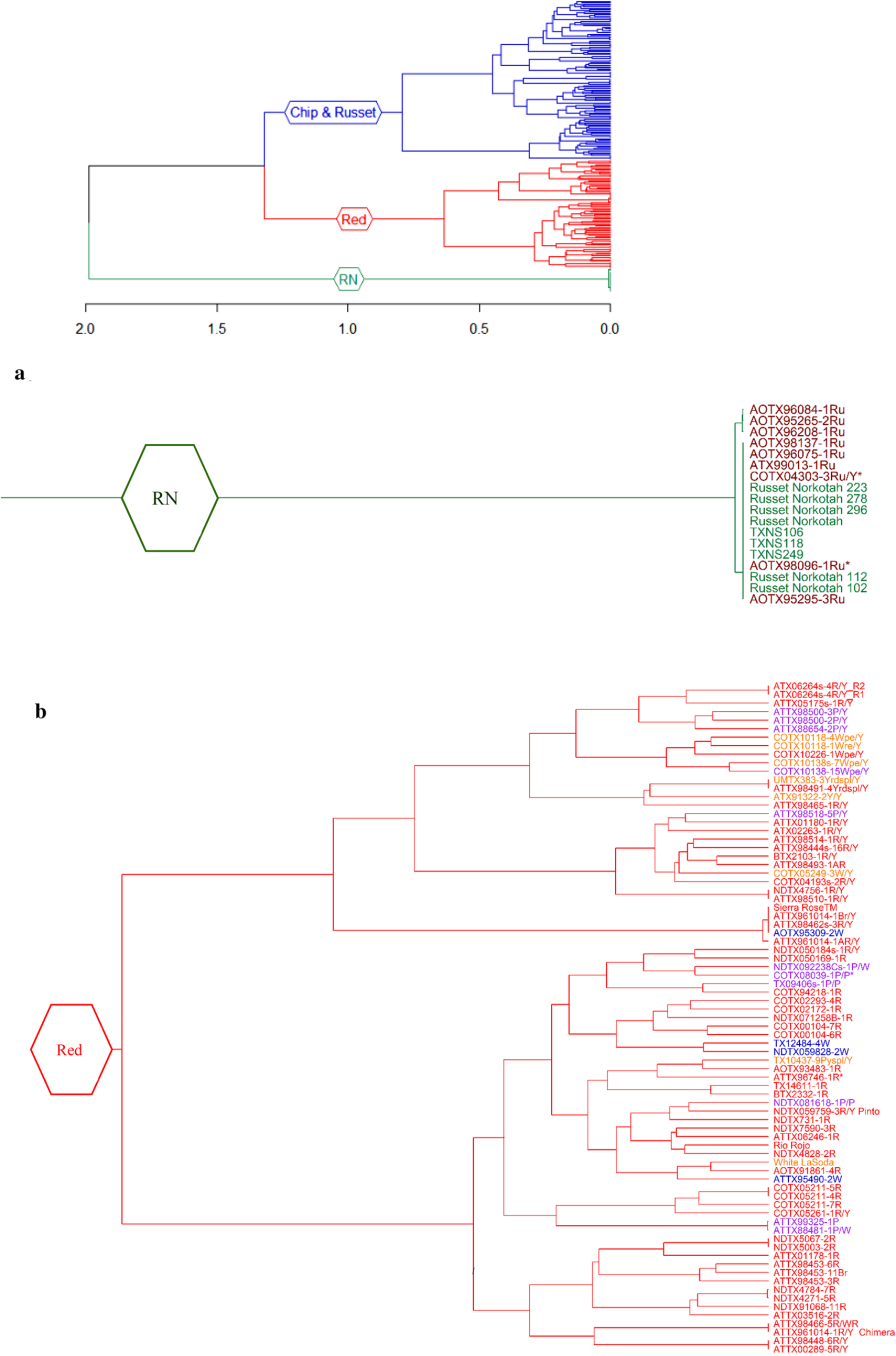

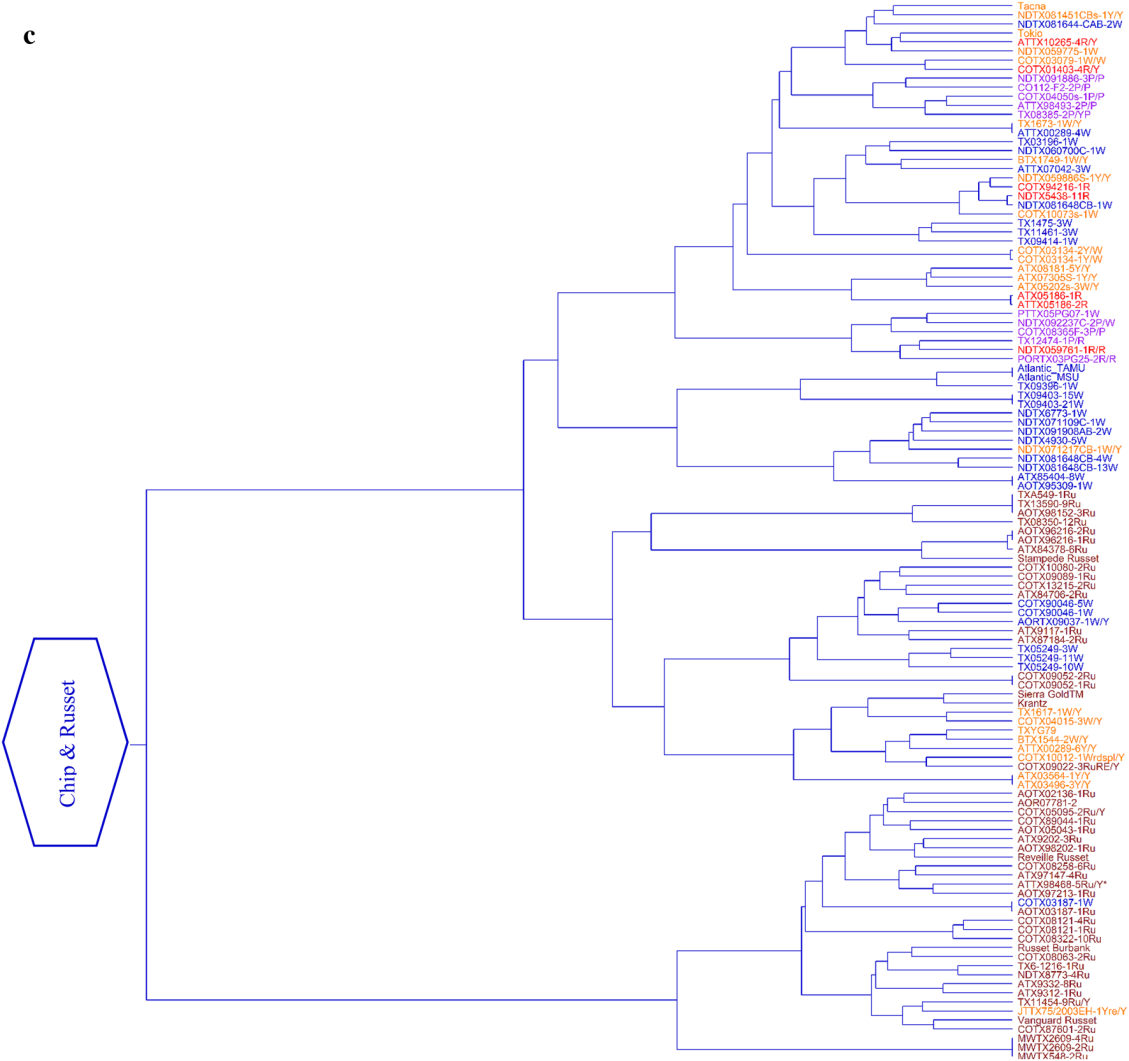
A Ward Dendrogram of the 214 clones using hierarchical clustering (method = “ward D”), the lower part (**a**) representing Russet Norkotah and its strains, the middle part (**b**) representing red and specialties and top part (**c**) representing chipping and russet clones are shown separately. In the X-axis are represented the Nei’s genetic distances between clones. The color of the clones represents the market class (Red = red clones, Purple = purple clones, and Yellow = yellow clones; Green = Russet Clones, and; Blue = chipping clones). Corrected names are indicated by an asterisk sign (*) at the end of the clone name. The plots were constructed using *Ape* R package^70^.

The dendrogram generated using Nei genetic distance and hierarchical clustering also revealed the presence of three clusters in the population (Fig. 6; Supplementary Information Table S8). The assignment of the clones to the groups in the dendrogram corresponded to 92% and 93% with the allocation made by the STRUCTURE and DAPC analysis, respectively. Groupings of the clones were observed based on their lineage/pedigree. As a result, selections with one or both common parents clustered together along with their parental clones in the same group. However, the clones were not separated based on cross location. Cluster 1 (18 clones) comprised mainly of Russet Norkotah, its eight strains (TXNS 106, TXNS 118, TXNS 249, Russet Norkotah 102, Russet Norkotah 112, Russet Norkotah 223, Russet Norkotah 278, and Russet Norkotah 296), and nine other russet clones (Fig. 6a). This is equivalent to 27% of the total russet clones used in this study. The dendrogram shows very low/no genetic distances between them. The origin of the clones in this cluster traces to crosses made by four breeding programs (Idaho, North Dakota, Colorado, and Texas). Similarly, cluster 2 (94 clones) comprised mostly of reds, yellows, and purple clones (Fig. 6b). This is equivalent to 96%, 40%, and 76% of the total red, total yellows, and total purple clones, respectively. In the cluster, Sierra Rose and four additional red clones were distinct from the remaining clones in the group. Four chipping clones (AOTX95309 − 2 W, ATTX95490 − 2 W, TX12484 − 4 W, and NDTX059828 − 2 W) appeared as exceptions in this cluster containing mainly red clones. The most prominent varieties in this cluster include White LaSoda, Sierra Rose, and Rio Rojo. The cross-location of the clones in this cluster traces to eight potato breeding programs in the United States. Lastly, Cluster 3 (102 clones) comprised of chipping clones, russets, yellows, and purple. This is equivalent to 90%, 73%, 59%, and 23% of the total chipping clones, total russets, total yellows, and total purple clones, respectively (Fig. 6c). Seven red clones appeared as exceptions in this cluster. COTX03187 − 1 W grouped with russets rather than grouping with chippers. The most prominent varieties in cluster 3 include Atlantic, Tacna, Tokio, Sierra Gold, Krantz, and a recently released Texas variety COTX09022-3RuRE/Y (russet skin red eyes and yellow flesh, released under the experimental name). The origin of the clones in this cluster traces to crosses originally made by eight potato breeding programs in the United States.

Under the current naming convention, the Texas Potato Breeding program typically uses a clone code that provides information about the place where the cross was made, where it was selected, year the cross was made, family number, selection number, type of skin, and sometimes type of flesh and other characteristics. For example, in ATX91137-1Ru, ‘A’ cross made in Aberdeen, Idaho, TX = selected in Texas, 91 = year cross was made, 137 = family number, -1 = selection number, and Ru = russet skin. After inspecting the dendrogram, we observed mislabeling in 5 clones (2.3% of the total clones). For instance, a russet clone AOTX98096 − 1Ru was mislabeled as a red clone AOTX98096 − 1R. Likewise, COTX04303-3Ru/Y was mislabeled as COTX04303-3R/Y. The SNPs grouped both of them with russets and Russet Norkotah strains in the dendrogram. Inspection of parentage gave a hint about the error and the minitubers produced in the greenhouse further confirmed that these should be russet clones (Supplementary Fig. S5). All the corrected names are reflected in the dendrogram with an asterisk sign (*) at the end of the name and name changes are listed in Supplementary Information Table S9. Atlantic was repeated (TAMU and MSU version) as quality control to detect duplications and they had almost zero Nei’s distance between them. After SNP comparisons, we found that some clones were identical. For instance, sister lines TX09403-15 W and TX09403-21 W cluster together and had almost zero Nei’s distance between them. Similarly, Russet Norkotah and Russet Norkotah strains could not be distinguished by the SNPs used in the current study. In another instance, clone AOTX95309-2 W did not group with sister line AOTX95309-1 W. AOTX95309-2 W clustered together with Reds and tubers were red. Based on the dendrogram, parentage, and tuber color, the clone AOTX95309-2 W could be considered an unintended mix and should be removed from the program. The use of SNP genotyping aided the discovery of typographic errors that occur during handling clonal material in the breeding program and/or tissue culture operations. Further, SNPs can also be used to define unique molecular fingerprints of released varieties and advanced clones and to calculate similarities (or distances) between new varieties and reference varieties and other released varieties.

### Identification of candidate loci under selection

**Figure 7:**
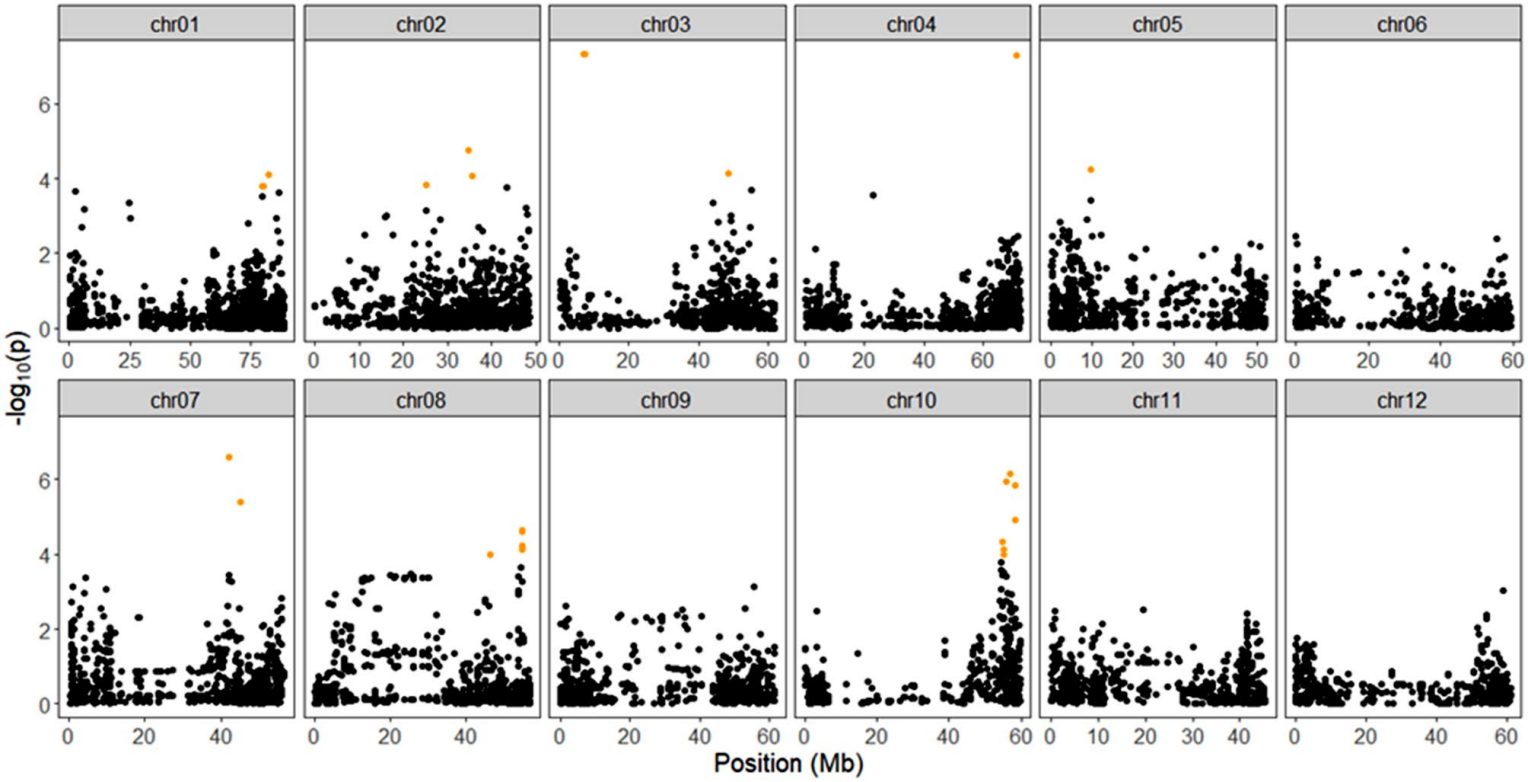
Manhattan plot showing the distribution of candidate outlier SNPs from PCAdapt^72^ where Y-axes represent P values and significant SNPs above α = 0.05 are in orange color.

**Figure 8:**
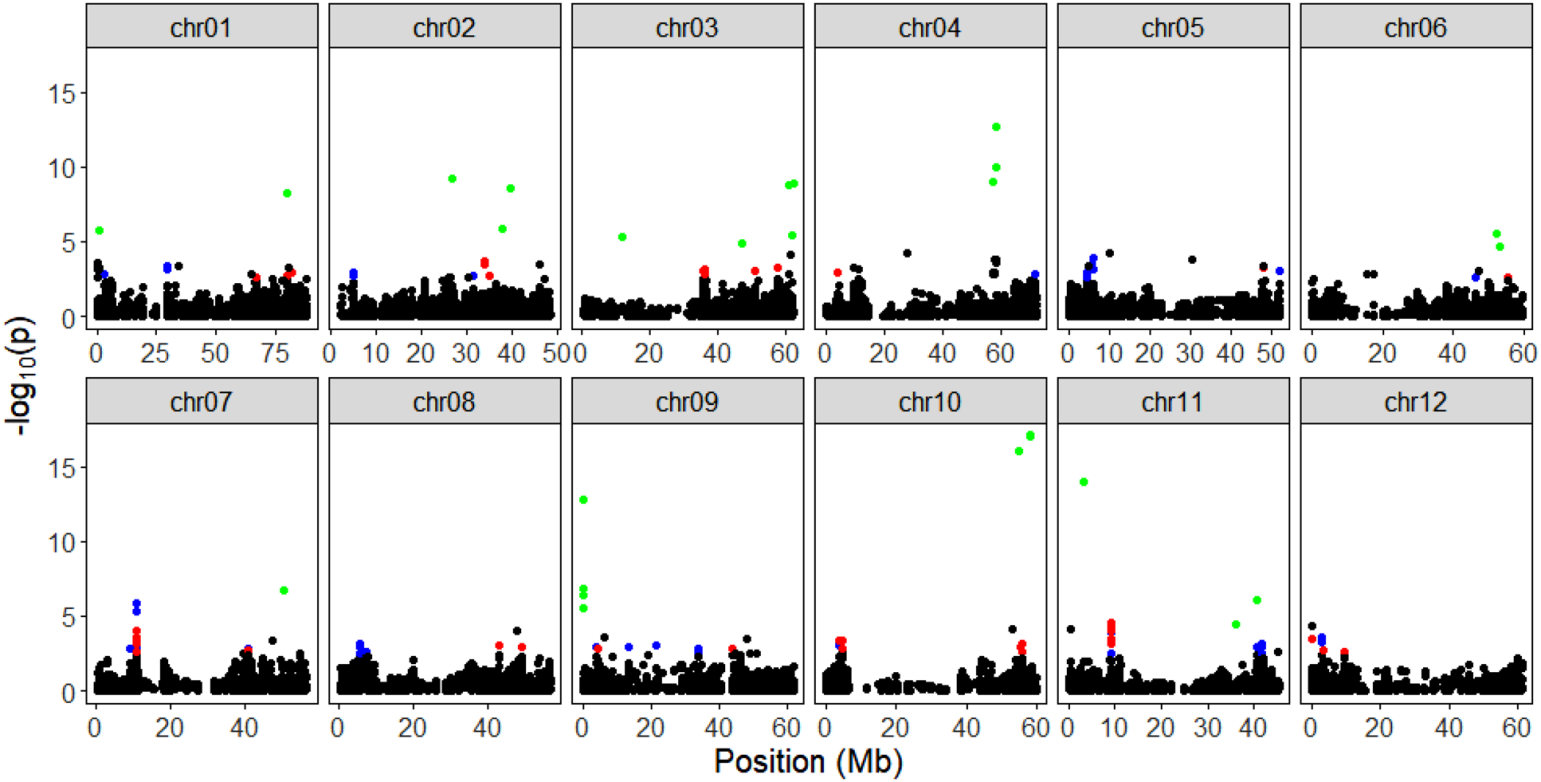
Distribution of standardized iHS scores in three groups of potatoes using rehh R package^75^. Significant SNPs above 1% false discovery rate (FDR) threshold are colored according to groups (iHS_chipru = blue; iHS_red = red; iHS_rn = green).

**Figure 9:**
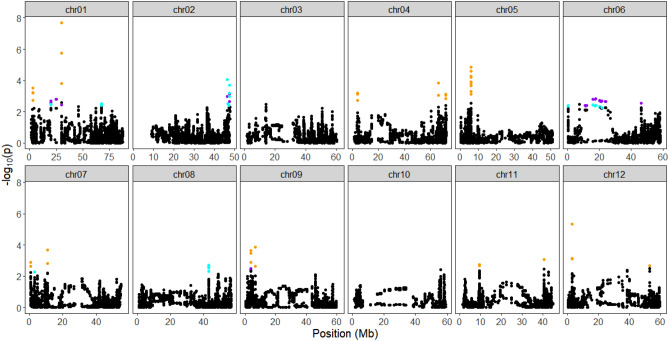
Distribution of standardized XP-EHH scores between three groups of potatoes using rehh R package^75^. Significant SNPs above 1% false discovery rate (FDR) threshold are colored according to groups (XP-EHH chirpru vs red = orange; XP-EHH chipru vs rn = purple; XP-EHH red vs rn = cyan).

Using the proportion of explained variance displayed, and projecting individuals on the principal components as a score plot (Supplementary Fig. S6), we estimated the optimal number of PCs from the SNP matrix to be three. At α = 0.05 corrected for the genomic inflation factor (λGC = 1.20), 26 SNPs were found under selection on chromosomes 1, 2, 3, 4, 5, 7, 8, and 10 using the PCA-based method (Supplementary Information Table S10). Some of the selected SNPs had known functions. For example, a SNP (PotVar0120627) was selected at 48.6 Mb on chromosome 3. It had been reported that the Y-locus controlling the white-to-yellow flesh color in potato mapped to chromosome 3^24^ and is believed to be regulated primarily by the b-carotene hydroxylase (*BCH*) gene^25^. Likewise, after adopting the false discovery rate of 0.01, 127 SNPs (Supplementary Information Table S11) and 100 SNPs (Supplementary Information Table S12) were found under selection using the iHS and XP-EHH tests, respectively. Figures 7, 8, and 9 shows the Manhattan plots illustrating the SNPs identified as being under selection pressure on all potato chromosomes according to the three tests assayed.

Eighteen regions were identified under selection by at least two of the statistics applied and were defined as candidate selection sweep regions. These regions occur on chromosomes 1, 2, 4, 6, 7, 8, 9, 10, 11, and 12 (Table 1). These SNPs were related to diversification and some of them were found associated with a specific phenotype. Some of the candidate genes had known functions, which are summarized in Supplementary Information Table S13 and partly discussed in the next section.

**Table 1:** Description of the candidate selective sweep regions detected using PCAdapt, iHS and XP-EHH analyses in potato.

Chr	Selective sweep region (Mb)	No. of candidate genes	Top significant SNP	Candidate gene pathway	Functional Annotation	Detection analysis	Max iHS/XPEHH/PCAdapt statistic	*P*-val
1	2.60–3.10	47	PotVar0044963		Flesh Color	iHS_chipru, XPEHH_chipru_red	3.1, 3.4	2.8, 3.2
1	19.41–19.91	11	solcap_snp_c2_49758			XPEHH_chipru_rn, XPEHH_red_rn	3.1, 2.9	2.7, 2.5
1	29.43–29.93	15	PotVar0122478			iHS_chipru, XPEHH_chipru_red	−3.5, − 5.6	3.4, 7.7
1	66.98–67.48	44	PotVar0098497			iHS_red, XPEHH_red_rn	−3.0, − 2.9	2.6, 2.5
2	46.15–46.65	68	solcap_snp_c2_24864	*Dof Zinc Finger Protein-StCDF3, CONSTANTS-CO*	Length of plant cycle and tuberization	XPEHH_chipru_rn, XPEHH_red_rn	3.3, 3.9	3, 4
4	3.66–4.16	37	PotVar0106879			iHS_red, XPEHH_chipru_red	3.2, −3.1	2.9, 2.7
6	15.52–16.02	12	solcap_snp_c2_18787			XPEHH_chipru_rn, XPEHH_red_rn	3.2, 2.9	2.8, 2.4
6	17.14–17.64	8	solcap_snp_c1_9601			XPEHH_chipru_rn, XPEHH_red_rn	3.2, 2.9	2.8, 2.4
6	20.06–20.56	14	solcap_snp_c2_56793			XPEHH_chipru_rn, XPEHH_red_rn	3.1, 2.8	2.7, 2.3
6	21.00–21.50	16	solcap_snp_c2_33233			XPEHH_chipru_rn, XPEHH_red_rn	3.1, 2.8	2.7, 2.2
6	21.48–21.98	8	PotVar0083629			XPEHH_chipru_rn, XPEHH_red_rn	3.1, 2.8	2.7, 2.2
7	10.58–11.08	20	solcap_snp_c1_2404		Stolon attachment	iHS_chipru, iHS_red	3.2, − 3.4	2.9, 3.2
7	40.36–40.86	25	solcap_snp_c2_9380			iHS_chipru, iHS_red	3.2, 3.1	2.8, 2.7
8	42.87–43.37	32	PotVar0086811			iHS_red, XPEHH_red_rn	3.3, − 2.8	3, 2.3
9	3.48–3.98	43	PotVar0012376		Flesh Color	XPEHH_chipru_red, XPEHH_chipru_rn	− 3.6, − 3.0	3.5, 2.5
10	55.60–56.10	57	PotVar0005291	*LOG3* gene, *Adenylyl-sulfate kinase*	Cytokinin metabolism, Pelargonidin	PCAdapt, iHS_red	30.5, 3.4	6.0, 3.2
11	9.12–9.62	28	solcap_snp_c2_53683			iHS_chipru, iHS_red	− 4.1, − 3.5	4.4, 3.3
12	2.83–3.33	54	PotVar0031150			iHS_chipru, XPEHH_chipru_red	3.7, 3.4	3.6, 3.2

### Core set identification

The analysis of genetic diversity and population structure of 214 clones identified sub-populations in the clone bank and some of the genotypes were quite similar. A core set of 43 clones (Supplementary Information Table S14) was selected to maximize diversity and minimize redundancy using Core Hunter 3 software. Among the core set, 14 clones were from the Chipping market class, 11 Russet, and seven, five, and six from the Red, Purple, and Yellow market classes, respectively. The genetic diversity of the core set was estimated to represent the extent of diversity captured from the total collection. Comparisons of all genetic parameters indicated that the values for the core set were almost equal to those for the total collection (Table 2). The mean genetic distance of the whole collection was 0.09, but this value increased to 0.10 in the core set. Similarly, the mean PIC and the mean MAF of the whole collection were both 0.31, while those of the core set were 0.31 and 0.30, respectively. DAPC analysis and hierarchical clustering showed the presence of four clusters in the core set (Supplementary Figs. S7 and S8).

**Table 2:** Comparison of the genetic diversity of the whole collection (214 clones) versus a core set (43 clones) based on genetic distance, polymorphism information content (PIC) and minor allele frequency (MAF).

Nei’s genetic distance				PIC				MAF			
Whole collection (214 clones)		Core set (43 clones)		Whole collection (214 clones)		Core set (43 clones)		Whole collection (214 clones)		Core set (43 clones)	
Mean	Range	Mean	Range	Mean	Range	Mean	Range	Mean	Range	Mean	Range
0.09	0–0.17	0.10	0.07–0.17	0.31	0.09–0.38	0.31	0.10–0.38	0.31	0.05–0.50	0.30	0.06–0.50

### Pedigree information

**Figure 10:**
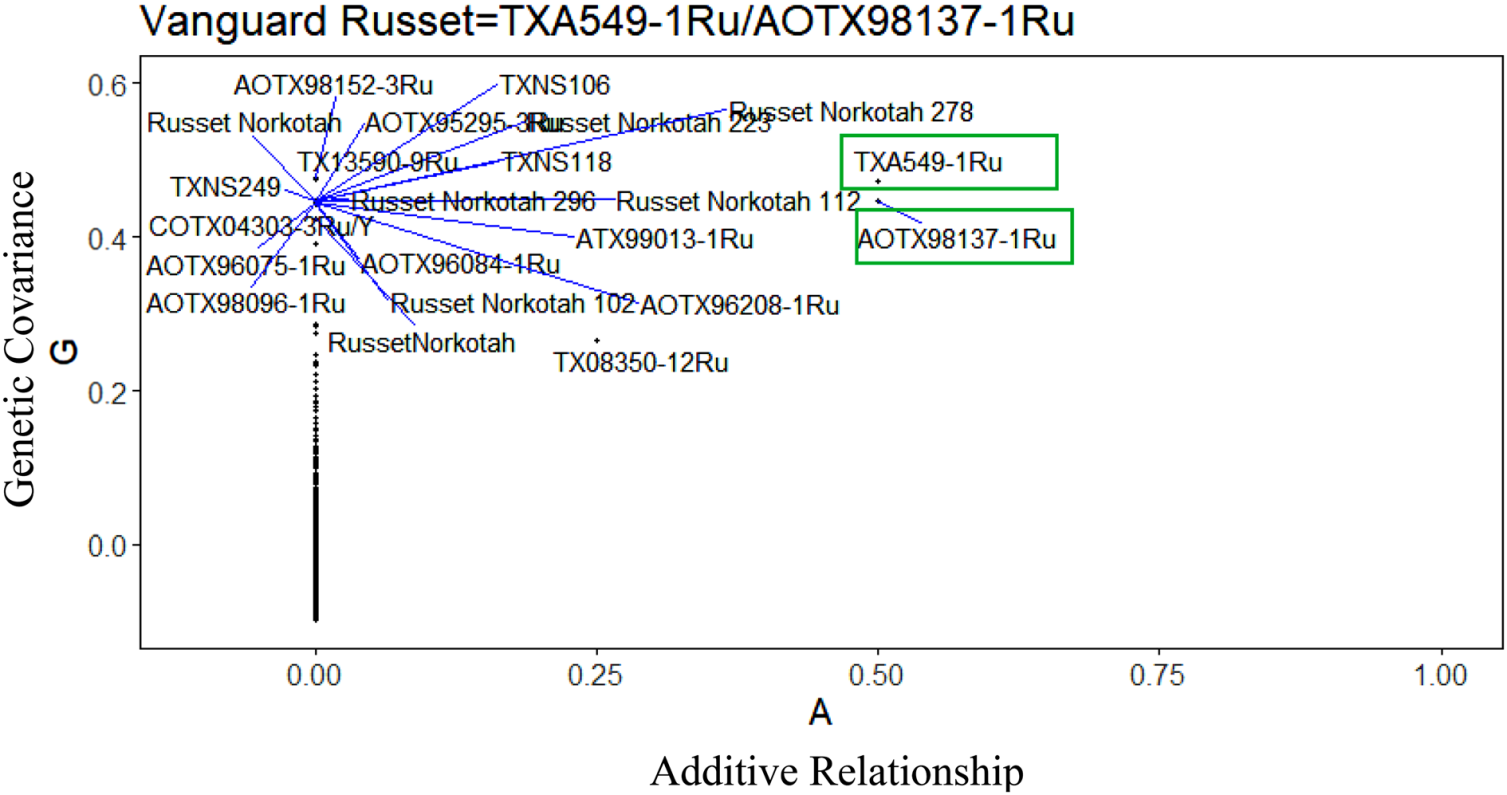
Population-wide comparison of genetic covariance calculated from markers with the additive relationship calculated from pedigree records, for Vanguard Russet using R software^82^.

For 12 of the parent–offspring trios having genotyping data for both parents, pedigree was found accurate for ten trios with no pedigree conflict. Figure 10 shows the marker vs. pedigree plot for Vanguard Russet without pedigree errors. The parents are in the top right corner of the figure, with both an additive relationship (A) and genetic covariance (G) of 0.5 and unrelated individuals with additive relationship A = 0 to the parents. One of the grandparents of Vanguard Russet, TX08350-12Ru is plotted at A = 0.25 and has less genetic covariance than the parents based on markers. Whereas, for clone NDTX4930-5 W and TX11461-3 W the conflict rate was 24.5% and 19%, respectively (Table 3). The male parent of NDTX4930-5 W and female parent of TX11461-3 W is found erroneous from the marker vs. pedigree plot. In the case of 12 clones with one parent genotyped, five parents were found correct, and seven parents were found erroneous (Supplementary Fig. S9–S11).

**Table 3:** Parent–offspring trios for which both recorded parents were genotyped. Pedigree conflict rates are shown in percentages.

Clone Name	Market Class	Female Parent	Male Parent	Pedigree Conflict %
Atlantic	Chipping	Wauseon	Lenape	0.0
NDTX4930-5 W	Chipping	ND860-2	**Western Russet**^**†**^	24.5
TX09396-1 W	Chipping	Atlantic	Lamoka	0.0
TX09403-21 W	Chipping	Waneta	Ivory Crisp	0.0
TX11461-3 W	Chipping	**Waneta**^**†**^	Chipeta	19.0
TX09403-15 W	Chipping	Waneta	Ivory Crisp	0.0
COTX08121-1Ru	Russet	AC96052-1Ru	Blazer	0.0
COTX08121-4Ru	Russet	AC96052-1Ru	Blazer	0.0
TX08350-12Ru	Russet	TXA549-1Ru	AC96052-1Ru	0.0
Vanguard Russet	Russet	TXA549-1Ru	AOTX98137-1Ru	0.0
COTX08322-10Ru	Russet	Blazer	AC96052-1Ru	0.0

^**†**^ The erroneous parent is shown in bold.

## Discussion

Tissue culture clone banks often contain potato varieties and breeding lines from several different regions and programs. Characterizing breeding collection germplasm is crucial in plant breeding, as the genetic advancement of economically valuable traits relies on the genetic diversity available within the breeding gene pool. Knowledge about genetic diversity also assists in minimizing the use of closely related clones as parents in breeding programs, which might lead to a high risk of inbreeding depression and reduced genetic variation. Genetic diversity, population structure, and molecular markers knowledge may accelerate the selection of desirable traits in potato. In the present study, population structure and genetic diversity were evaluated in a tissue culture clone bank collection composed of 214 diverse clones. This collection contains advanced selections from the TAMU Potato Breeding Program entered in tissue culture over 40 years of breeding efforts.

The availability of SNP arrays has enabled germplasm genotyping of crops like potatoes. SNP distribution across the genome assessed by analyzing filtered SNP density shows the typical pattern of distinctly reduced recombination in pericentromeric regions and increased varying recombination rates in euchromatic regions for all chromosomes. Larger regions with no SNP coverage are usually found in large pericentromeric regions, where repetitive DNA makes it difficult to distinguish unique flanking regions around SNPs^26,27^. A similar distribution has been observed in Sorghum^28^, wild tomatoes^29^, and *Prunus*^30^. SNPs offer high-resolution markers to breeding programs far beyond traditionally used approaches depending solely on pedigrees^31^ or phenotypic data^32^. In the present study, the average PIC value was 0.31. Most SNPs (69%) had PIC values ranging from 0.30 to 0.38, while for the remaining percentage, it was < 0.30. The SNPs having PIC values ranging from 0.25 to 0.5 are considered moderately informative^33^. This may support the idea that breeding efforts, genetic diversity in our set of clones has not been narrowed. Similar PIC values were previously reported in collections of potatoes tested for genetic diversity using the SolCap SNP array^10,34^.

Heterozygosity is an indicator of genetic variability in a population and it is related to the polymorphic nature at each locus. In this study, a high level of heterozygosity was observed; in the TAMU potato breeding collection, this could be due to the high levels of genetic variation at loci with vital significance for adaptive response to environmental changes. Loss of heterozygosity was related to lower fitness^35^. Potato is an outcrossing species; thus, heterozygosity is usually higher than expected. Selection, migration, mutation, hybridization, polyploidization, and introgression elucidate the high diversity of potatoes^10^. The average percent heterozygosity (59%) observed in the TAMU potato collection was similar to Hirsch et al.^7^ and Igarashi et al.^12^, who reported the average value of 56% and 60%, respectively. However, the lowest heterozygosity value we observed was 22% that of ATX91322-2Y/Y, which was found to be a diploid selected by the program based upon five cluster calling. Igarashi et al.^12^ reported that the average percent heterozygosity of the 2 × varieties (23%) was much lower than that of the 4 × genotypes. Five clusters genotypic calling has successfully been used to predict ploidy determinations of diploid, triploid, and tetraploid samples^11,36^. When the simplex and triplex frequency was close to zero, the sample was considered to be diploid, and when the frequency was over 0.20, then the progeny samples were considered to be tetraploid. This finding is similar to Hirsch et al.^7^, indicating that greater ploidy can be correlated with greater heterozygosity, and vice versa. Almost all the highly heterozygous clones had negative inbreeding coefficients, which happen when observed heterozygous clones are larger than expected due to an excess outcrossing. Increasing the heterozygosity of clones and widening their genetic base are important aspects of breeding programs to have desired combinations of abiotic and biotic stress tolerances and high yield. It is clear from the heterozygosity analyses and SNP evaluation, that the Texas A&M potato breeding program harbors considerable genetic diversity.

Tajima’s D^37^ provides a distinction between randomly altering loci and non-randomly evolving loci arising from the directional selection, introgression, genetic bottleneck, and/or drift. Generally, a positive value of Tajima’s D arises from an excess of intermediate frequency alleles and can result from population bottlenecks, structure, and/or balancing selection^38^. These factors are likely present in potato breeding clones. The observed Tajima’s D value of 4.29 in this study is higher than in sorghum (0.30)^39^ and soybean (1.08)^40^, both of which show a significant bottleneck in their population history. To elucidate the possibility that the elevated D value occurred due to population subdivision, we assessed population structure as suggested by Pritchard et al.^41^. When SNPs from all chromosomes were included in the analysis, a significant subdivision was observed (Fig. 4) indicating a relatively heterogeneous population.

Understanding population structure helps allow the successful use of genotypes for breeding purposes. Alleles that are divergent among clusters are a guide to detecting the principal differences due to breeding strategies and different origins among subpopulations. The STRUCTURE analysis provided further insight into the admixture and the number of populations in this collection. Structure analysis identified subgroups, as in other studies^7,9^. Our results support hybridization or outcrossing among the individuals and a five percent admixture.

DAPC analysis divided the population into well-defined clusters according to their genetic structure and market classes. The DAPC approach offers an alternative to STRUCTURE software as it does not require the populations to be in Hardy–Weinberg equilibrium, and it can support large sets of data^42^. Our results identified good consistency between STRUCTURE and DAPC analysis when admixed clones were not considered.

The clustering of individuals gives interesting cues for increasing diversity in breeding programs and germplasm collections. Clear knowledge of the germplasm structure and clusters assists in parental choice in breeding programs, improving genetic diversity, and enhancing the potential gain from the selection. Both help to increase breeding program efficiency to face new demands from consumers and the industry, as well as new ecological issues like adaptation to climate change and pest resistances. In this study, clustering corresponded with the similarity in the genetic background of the clones. However, clustering was not found depending on the place where the original cross was done suggesting a huge gene flow across breeding programs due to the reciprocal exchange of true potato seed of unselected families and use in crossing blocks of parental germplasm from potato breeding programs throughout the United States. Bali et al.^34^ were unable to separate Russet potato clones according to the breeding programs they originated from, which was an indicator of the free-flow of germplasm among the potato breeding programs. Several quantitative differences (e.g. vine size, maturity, average tuber size, yield, etc.) existed among the strains and Russet Norkotah^43^. However, clonal selections (strains) were not differentiated genetically despite using more than 11,000 genome-wide SNP markers. Previous studies using Amplified Fragment Length Polymorphism (AFLP) and microsatellite marker were also unable to detect differences between intraclonal variants of the potato cultivar Russet Norkotah^44^. The differences between the strains could be due to epigenetic variation, and most of them may not be observed by SNPs^45^.

Maintaining consistent and unique clone names in the clone bank is important for future cultivar identification, research, and breeding. There are many instances in the program where naming errors could be introduced. The longer a clone is handled in the program, the greater the potential of mixing or mislabeling. The Infinium 22 K V3 Potato SNP Array generated unique genetic fingerprints to identify accessions where errors had occurred. The majority (97.6%) of the clones evaluated had no errors in genetic identity. It is common in most gene banks to have some errors in the collection. Ellis et al.^11^ at the International Potato Center (CIP) found 4.4% of accessions were genetically mismatched, and in some cases, the SNP results identified the mixed accession. Barcoding, automatic data collection, curation, and other quality control strategies will help to minimize errors. Studies such as this can help identify and correct errors in the breeding program. In addition, SNP fingerprints and genetic distance comparisons can be useful for plant variety protection (PVP), as well as for the verification of the identity of clones in the foundation, certification, and breeding programs.

Our main goal for selection signature analysis was to detect regions that show preferential selection in the genome of potatoes. To accomplish this, we used three different, but complementary, statistical methods: PCAdapt, iHS, and XP-EHH. The use of a combination of methods for selection sweep detection allows different emerging patterns of selection to be identified, and it also improves the reliability and accuracy of the analyses^46^. Potato breeding efforts currently center on improving different market classes such as chip and French fry processing, pigmented, table russets, and yellows^47^. Most (but not all) hybridizations are made between clones within a market class. Over time one might expect these market classes to diverge, not only in terms of the few traits that define each class, but also in terms of unlinked, selectively neutral DNA markers^47^. Several of the identified SNPs and sweep regions in this study are associated with functions of interest and warrant further investigation.

A SNP (PotVar0120627) was selected by PCAdapt at 48.6 Mb on chromosome 3 controlling the white-to-yellow flesh color. Sharma et al.^48^ using genome-wide association mapping had also found a strong association for flesh color at 49.4 Mb on the same chromosome. Similarly, SNPs belonging to the sweep region detected by this study on chromosomes 1 and 9 (Table 1) had been previously identified as significant SNPs for flesh color^48^. The SNPs at the sweep region (10.58–11.08 Mb) on chromosome 7 were reported to have a significant association for the stolon attachment trait in potato^48^. In a previous QTL study, Manrique-Carpintero et al.^35^ identified candidate genes (*Dof Zinc Finger Protein-StCDF3, CONSTANTS-CO*) in the photoperiod regulatory pathway associated with length of plant cycle and tuberization in the QTL region on chromosome 2 around 46 Mb. The XPEHH test from our study has also detected a sweep at the same location (46.15–46.65 Mb) on the same chromosome (Supplementary Information Table S12). Likewise, Manrique-Carpintero et al.^35^ had reported a *cytokinin riboside 5′-monophosphate phosphoribohydrolase LOG3* gene location in the QTL region at 56 Mb on chromosome 10. Differential expression and pleiotropic effects of LOG genes show their major role in cytokinin metabolism to modulate plant growth and development in Arabidopsis^49^ and tomato^50^. The sweep region at chromosome 10 (55.60–56.10 Mb) detected by both iHS and PCAdapt test in our study matched with this finding. In the potato *Sucrose transporter 4 (SUT4)* gene involving an accumulation of sucrose and starch in the terminal sink, organs are located at 65.8 Mb on chromosome 4^35,51^. Our XPEHH test between the ChipRu and Red groups selected three SNPs (solcap_snp_c2_55781, solcap_snp_c2_55780 and solcap_snp_c2_55779) at 65.9 Mb on the same chromosome. It is well known that Red potatoes have less starch and more sugars than Chip and Russet potatoes^52^. Parra-Galindo et al.^53^ reported the QTL AnthoX_Adeny, colocalized on chromosome 10 at 57.3 Mb (PGSC0003DMT400060833/*Adenylyl-sulfate kinase* gene), explaining 41.1% of the phenotypic variance of pelargonidin. As illustrated in a colored potato study, the red cultivars contained predominantly pelargonidin derivatives, while the purple/blue varieties had peonidin, petunidin, and malvidin as the main aglycones^54^. These results will allow a better understanding of the genetic architecture and will open avenues for studying candidates for biochemical and functional studies of admixed advanced potato selections.

Most plant breeders want to make better use of plant genetic resources in their breeding programs but have trouble maintaining many clones and prioritizing clones for parental selection. Some breeders define a subset of clones that reflect the greater collection. The core subset can also be maintained as a backup collection to preserve important genes. In this study, we proposed a core set of 43 potato clones, accounting for 20% of the total collection using CoreHunter software. A sampling percentage of 20 ~ 30% was suggested by Hintum et al.^55^. Nevertheless, all core germplasm sets do not have a fixed size, as different crops and targets require different sampling percentages. In earlier studies, a core set of 48 was defined for capturing the genetic diversity of a collection of 350 tetraploid cultivated potato varieties by using simple sequence repeats (SSR) data^56^. A core set of 27 genotypes was developed from 138 accessions of potato cultivars from the Western Highlands region of Cameroon using SSR markers^57^. Core Hunter software has been used for core set selection in earlier studies of different crops, including wheat^58^, Brazilian grapevine germplasm^59^, banana^60^, sweetpotato^61^, and common bean^62^. In many reports, genetic diversity and cluster analysis were used to evaluate the efficiency of the development of the core germplasm set. In the present study, the genetic distance increased as expected after the removal of genetically similar clones during core germplasm set development. Having a core collection as a backup of important genes and source of parents is a good idea. However, in reality, core collections may not meet the needs of modern breeding approaches, such as genomic selection and genome-wide association studies where more individuals are desired to increase the statistical detection power.

In conclusion, our analysis of the genetic diversity and population structure of the advanced clones in the TAMU Potato Breeding Program found a significant subdivision among clones, indicating a heterogeneous collection. Further, the SNP markers used in the study allowed the differentiation among breeding clones and the development of a core germplasm set of 43 clones, accounting for 20% of the total collection. Additionally, we used the SNP array to validate pedigree information. The genome-wide SNP characterization of these 214 clones, development of the core set, and reporting of the correct pedigree in this study will be useful for future genomic studies, parental selection, and germplasm management in potato breeding program.

## Methods

### Plant material

Two hundred fourteen potato clones were included in this study (Supplementary Information Table S1). The clones represent fresh and processing market classes with a variation for skin type (russet and smooth), flesh and skin color, shape, agronomic, biotic, abiotic, and quality traits. The collection comprised 31 chipping, 62 russet, 32 yellow-skinned, 68 red-skinned, and 21 purple-skinned clones. The collection was initiated during the 1980s and consisted mainly of early generation and advanced clones selected by the TAMU Potato Breeding Program. The introduction into tissue culture and virus eradication of early and advanced potato selections is a regular practice in the TAMU Potato Breeding Program since disease-free stocks from Texas selections are typically transferred to Colorado State University to produce clean seed for regional trials (SW and W). Some commercial cultivars developed by the TAMU Program were also preserved in the clone bank, including Russet Norkotah clonal selections (Russet Norkotah 112, Russet Norkotah 223, Russet Norkotah 278, and Russet Norkotah 296), Sierra Rose (ATTX961014-1R/Y), Sierra Gold (TX1523-1Ru/Y), Rio Rojo, COTX09022-3RuRE/Y (released under experimental ID), Reveille Russet (ATX91137-1Ru), Vanguard Russet (TX08352-5Ru) and Stampede Russet (TXAV657-27Ru). Commercially popular varieties, including Russet Norkotah (standard), Atlantic, and Russet Burbank were included as reference genotypes. White LaSoda (a white skinned mutant of Red LaSoda selected by the TAMU Program) and Yukon Gold Strain (TXYG79) were also included in the study. All of the clones are now maintained in the TAMU breeding clone bank. To micropropagate the clones, tissue culture media consisting of Murashige and Skoog (MS) (4.8 g/L), sucrose (30 g/L), and agar (8 g/L) was used. Clean (disease-free status confirmed by ELISA assays) plant materials were multiplied and moved to the greenhouse in Fall 2018 to produce mini tubers. In the greenhouse, 12 plantlets of each clone were grown for 110 days in a standard flat insert (TO Plastics, MN, USA) (26.82 cm × 53.49 cm) with 32 cells (each cell size: 4.04 cm × 2.92 cm × 5.72 cm) filled with Sunshine Mix #1 (Sungro, Agawam, MA) with starter fertilizer Osmocote (Scotts Miracle-Gro, Marysville, OH). The photoperiod (light: dark) was 16:8 until flowering and 12:12 afterward to enhance tuberization. The greenhouse temperature averaged 20 °C with a minimum of 14 °C and a maximum of 31 °C. Minitubers were harvested in Spring 2019 and stored at room temperature for approximately a week to confirm the skin and flesh color of the clones.

### DNA extractions

Genomic DNA was extracted from 50 to 80 mg of fresh young potato leaves from tissue culture plantlets using the DNeasy Plant Pro Kit (Qiagen, Valencia, CA, USA). DNA quality was examined using 1% agarose gel 1X TBE (Tris–Borate and ethylenediaminetetraacetic acid) and staining with GelRed (Biotium Inc., CA, USA) using a V.U.V. transilluminator (Benchtop VUV Transilluminators, UVP). Quantification of DNA was performed in a spectrophotometer (Nano Drop, Thermo Scientific, Waltham, Massachusetts, USA). DNA concentration was verified using the Quant-iT PicoGreen dsDNA Assay Kit (Invitrogen, SanDiego, CA) and samples with uniform DNA concentration (50 ng μL^−1^) were prepared.

### SNP genotyping

Samples were assayed using the Infinium 22 K V3 Potato Array on the Illumina iScan (Illumina Inc., San Diego, CA, USA) at Michigan State University. V3 Potato array includes the SNP from the Infinium 8303 Potato Array with additional markers from the Infinium high‐confidence SNPs (69 K)^47^ and the SolSTW 20 K array^9^. Samples were SNP genotyped using the Illumina GenomeStudio 2.0.4 software (Illumina, San Diego, CA) for five-cluster (nulliplex = AAAA, simplex = AAAB, duplex = AABB, triplex = ABBB, and quadruplex = BBBB) marker calling using a custom tetraploid cluster file based on the PolyGentrain polyploid module calling of reference tetraploid samples (Illumina, San Diego, CA). The SNP genotype data were filtered to exclude low-quality, monomorphic SNPs, and loci with ≥ 10% missing data. Also, the alleles-design option was displayed in GenomeStudio to get genotypes in nucleotide format for STRUCTURE input. The genotyping data were transformed into diploid form as AAAA = AA, BBBB = BB, and AAAB, AABB, ABBB = AB to use in analysis packages which do not support polyploid data.

### Genetic diversity

SNP genotypic data were used to study genetic diversity and to understand the genetic relationship among clones. Allele frequencies, polymorphic information content (PIC), heterozygosity, and inbreeding coefficient were calculated in *snpReady*^63^ package in R using the diploid genotypic calls.

The average pairwise divergence among genotypes, which represents the nucleotide diversity per bp, π (pi), and the expected number of polymorphic sites per nucleotide, θ (theta), were estimated in TASSEL v5.2.39^64^ using the default settings for the diploid genotypic calls. The normalized measure of the difference between the observed (π) and expected (θ) nucleotide diversity, known as Tajima’s D, was also computed in TASSEL.

### Population structure

Population structure was determined using STRUCTURE software version 2.3.4^65^ using an admixture model of the diploid genotypic calls. STRUCTURE places clones in subpopulations based on similar patterns of variation. For each dataset, three replicates were performed for each value of K from one to ten with a 50,000 burn-in time, and the number of Markov Chain Monte Carlo replicates also set to 50,000. For each K, we checked whether the run parameters (likelihood, posterior probability of data and alpha) reach convergence within the burn-in period. The most probable K-value was determined by STRUCTURE Harvester^66^, using the log probability of the data [LnP(D)] and delta K (ΔK) based on the rate of change in [LnP(D)] between successive K-values.

### Discriminant analysis of principal components (DAPC)

Was done using the *adegenet* package^67^ in R to identify and describe clusters based on genetic relationships using a diploid form of genotyping data. The feature *find.clusters* was used to identify the number of clusters within the population. The K-means clustering decomposes the variable’s total variance into between-group and within-group components. The lowest associated BIC had defined the best number of subpopulations. The correct number of principal components (PCs) to be maintained was verified using a cross-validation feature (*Xval.dapc*). In this analysis, the data is divided into two sets: a training set (90 percent of the data) and a validation set (10 percent of the data). The members of each group are chosen by stratified random sampling, ensuring that at least one member of each group or population is reflected in the original data in both training and validation sets. DAPC is performed on the training set with a variable number of retained PCs, and the degree to which the analysis can accurately predict group membership of excluded individuals (those in the validation set) is used to determine the optimum number of retained PC. The sampling and DAPC procedures are repeated many times at every PC retention level. The best number of PCs that should be taken is associated with the lowest root mean square error. *SNPZIP* analysis was used to identify alleles with the largest contributions to form the linear discriminants and allocate the genotypes to the clusters. The coefficient of genetic differentiation among groups (F_st_) was calculated using *stamppFst* in StAMPP package^68^ in R.

### Hierarchal clustering

Pairwise Nei genetic distance^69^ was calculated, and a distance matrix was obtained with the StAMPP package^68^ using the tetraploid SNP genotype calls. The resulting matrix was used to build a dendrogram using the hierarchical clustering (method = “Ward D”) implementing in the *Ape* package^70^ in R. Duplications, mislabeling, and errors with the naming were identified from the dendrogram based on clustering. After removing duplicates and mislabeled clones, a core set of clone bank collection was developed for long-term in-vitro maintenance.

### Identification of selection signatures

Signatures of selection analyses were performed using 10,106 SNPs applying three complementary statistical methods. The outlier test PCAdapt^21^ was based on allele frequency differentiation whereas, the iHS^71^ (Voight et al., 2006) and the XP-EHH^22^ were based on linkage disequilibrium (LD) patterns.

PCAdapt Version 4^72^ was used to identify loci related to diversification in R. The option for performing LD clumping was applied, this removes variants in LD and ensures that more PCs capture population structure instead of LD structure^72^. The initial number of PCs was set as K = 20, and the scree plot was used to pick the K that explains much of the variance. The choice of K was also verified by projecting individuals on the principal components (called PCAdapt's score plot) to see if the clustering level was consistent with the value selected for K. The Mahalanobis distances were then used to search for outlier SNPs and transformed into p‐values to perform hypothesis testing. A Q-Q plot of the predicted p-values vs. observed p-values was used to visualize the distribution of the p-values. The cut‐off for identifying selections was then based on the q‐value method using the qvalue R package^73^, using 5% as false discovery rate threshold.

SHAPEIT2^74^ with a window size of 1 Mb and 500 iterations, including 200 burn-in and pruning iterations, was used to derive haplotypes for iHS and XP-EHH analyses. The iHS and XP-EHH analysis was done using the rehh package^75^ in R software. To allow better visualization and analysis of regions under selection, the iHS and XP-EHH scores were standardized to a distribution with zero mean and unit variance. In addition, p-values were calculated with the threshold set at 1 percent, as defined in Gautier and Naves^76^ and FDR performed following Storey and Tibshirani^77^. Candidate selection sweep regions were classified as SNP regions identified as being under selection by at least two of the statistics applied. Genes spanning 250 kb upstream and downstream of the candidate selection regions were retrieved from the representative gene annotation for the pseudomolecules from the Potato Genome Sequencing Consortium (PGSC) public data^23,78,79^ retrieved from http://solanaceae.plantbiology.msu.edu/pgsc_download.shtml.

### Core set identification

A core set of most diverse clones was identified using Core Hunter 3^80^. This software generates subsets based on multiple genetic measures, including both distance measures and allelic diversity indices (http://www.corehunter.org). The function *sampleCore* was run on a precomputed Nei’s distance matrix of 214 clones.

### Pedigree information

A curated dataset was used to check the accuracy of pedigree records using the methodology of Endelman et al.^81^. Pedigree information was assembled from variety release publications, published potato pedigree database, and TAMU potato program breeding records. If both parents were genotyped, the pedigree conflict rate was used to identify pedigree errors. For each of the parent–offspring trios (two parents and one offspring) in the dataset, a pedigree conflict metric was calculated as the percentage of monomorphic (i.e., non-segregating) markers in the cross at which the genotype of the offspring was different. When only one parent was genotyped, the marker vs. pedigree plot was used to confirm (or not) the known parent.

## Supplementary Information

Supplementary Information 1.

Supplementary Information 2.

Supplementary Information 3.

Supplementary Information 4.

Supplementary Information 5.

Supplementary Information 6.

Supplementary Information 7.

Supplementary Information 8.

Supplementary Information 9.

Supplementary Information 10.

Supplementary Information 11.

Supplementary Information 12.

Supplementary Information 13.

Supplementary Information 14.

Supplementary Information 15.
