# Supplementary material for: Genetic diversity and population structure of advanced clones selected over forty years by a potato breeding program in the USA
Source: Sci Rep. 2021 Apr 16;11:8344. doi: 10.1038/s41598-021-87284-x (PMC8052460; doi:10.1038/s41598-021-87284-x)
Supplement: Supplementary file 15 — Supplementary Information 15. [file 41598_2021_87284_MOESM15_ESM.docx]

**Genetic diversity and population structure of advanced clones selected over forty years by a potato breeding program in the USA**

Jeewan Pandey^1^, Douglas C. Scheuring^1^, Jeffrey W. Koym^2^, Joseph Coombs^3^, Richard G. Novy^4^, Asunta L. Thompson^5^, David G. Holm^6^, David S. Douches^3^, J. Creighton Miller Jr.^1,†^, and M. Isabel Vales^1,*^

^1^Department of Horticultural Sciences, Texas A&M University, College Station, TX 77843, USA

^2^Texas A&M AgriLife Research and Extension Center, Lubbock, TX 79403, USA

^3^Department of Plant, Soil, and Microbial Sciences, Michigan State University, East Lansing, MI 48824, USA

^4^USDA-Agricultural Research Service, Small Grains and Potato Germplasm Research, Aberdeen, ID 83210, USA

^5^ Department of Plant Sciences, North Dakota State University, Fargo, North Dakota 58108, USA

^6^San Luis Valley Research Center, Department of Horticulture and Landscape Architecture, Colorado State University, CO 81125, USA

† Deceased

*Corresponding Author: M. Isabel Vales, Ph.D., Department of Horticultural Sciences, 2133 Texas A&M University, College Station, TX 77843-2133; email: [isabel.vales@tamu.edu](mailto:isabel.vales@tamu.edu); Phone: 541-740-4437; ORCID id: 0000-0002-6895-3137

**Supplementary information**


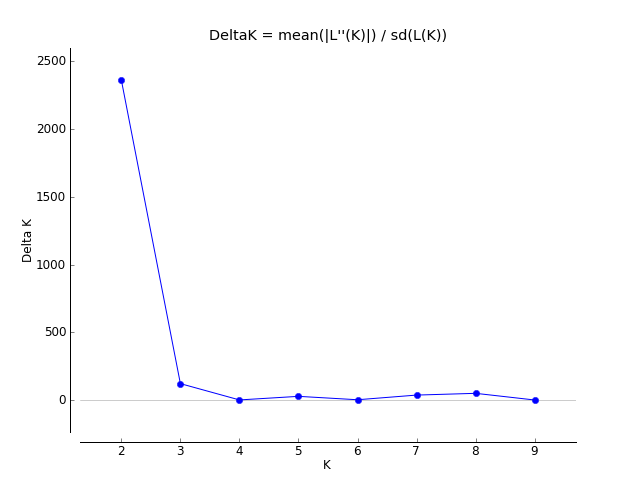


Number of populations

**Figure S1.** Estimation of the number of populations using LnP(D) derived Δk for K from 1 to 10 using 10,106 SNPs. The maximum of adhoc measure ΔK determined by structure harvester was found to be K = 3. The red arrow in the graph indicates the clear inflection point.


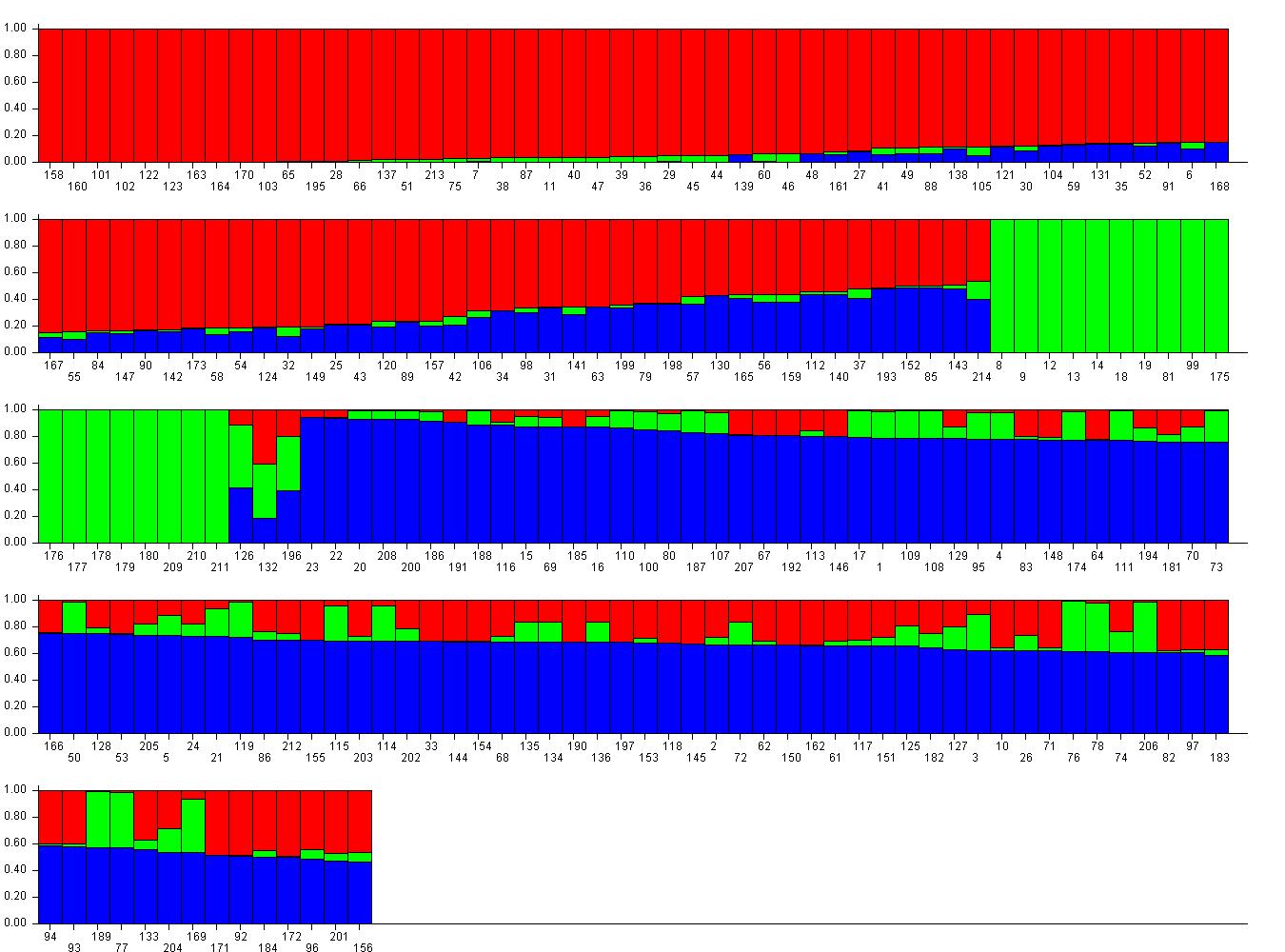


**Figure S2.** Model-based clustering using software STRUCTURE showing individual genotypes (x-axis) in the population structure of 214 potato clones (K = 3, red, green and blue) based on 10,106 SNPs. The y-axis indicates the subpopulation membership (relative scale 0-1). Individual potato genotypes are shown in the x-axis (the link between clone numbers and experimental identification is shown in Supplementary Table S5).


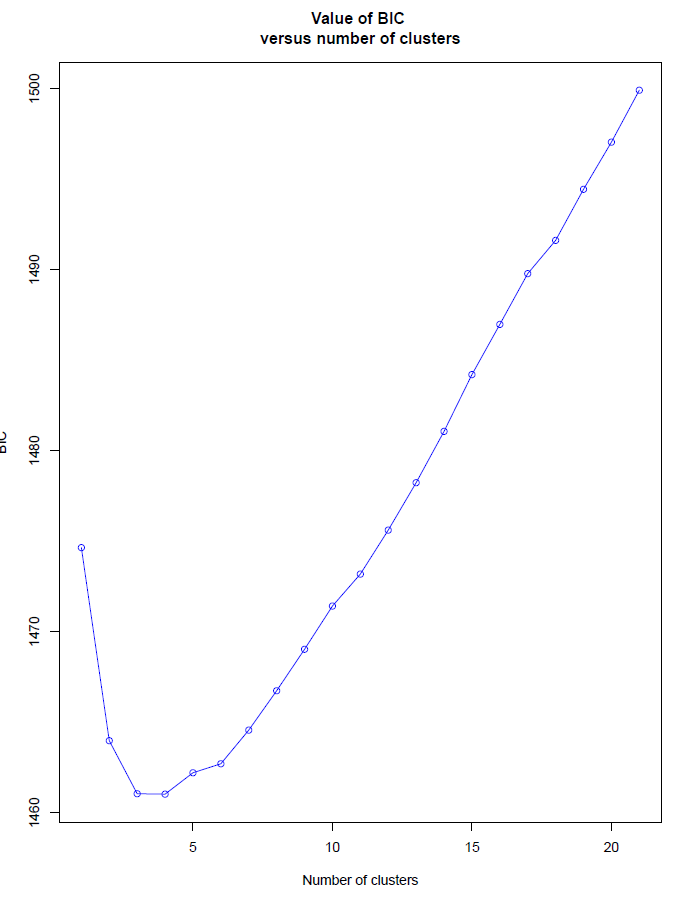


BIC

**Figure S3.** The lowest Bayesian information criterion (BIC) value was obtained using *find.clusters* function using *adegenet* R package^67^ for discriminant analysis of principal components (DAPC) analysis using 10K SNPs in the population of 214 potato clones.


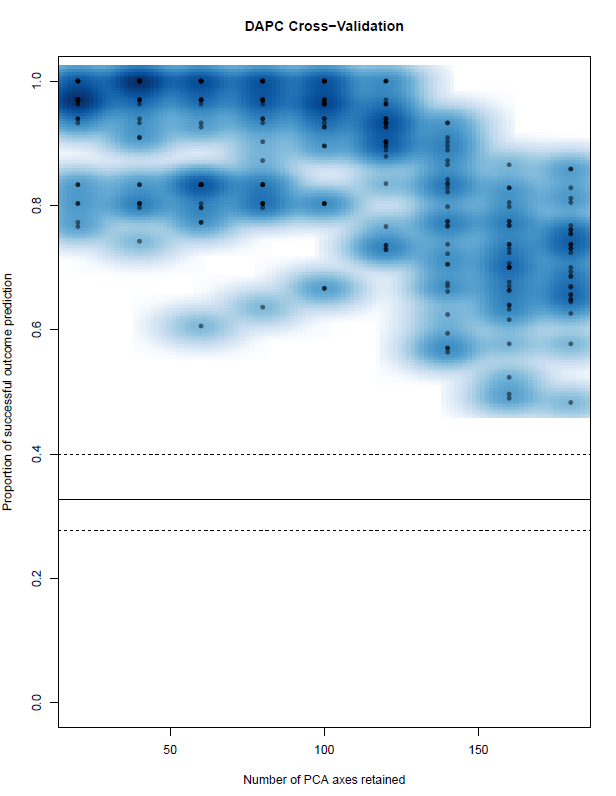


**Figure S4.** Cross-validation plot to guide the selection of the number of principal component axes (PCA) to retain a Discriminant Analysis of Principal Components Analysis using *adegenet* R package^67^. The PCA value that maximizes the proportion of successful outcomes and minimizes the mean square error (MSE) is 20.

**
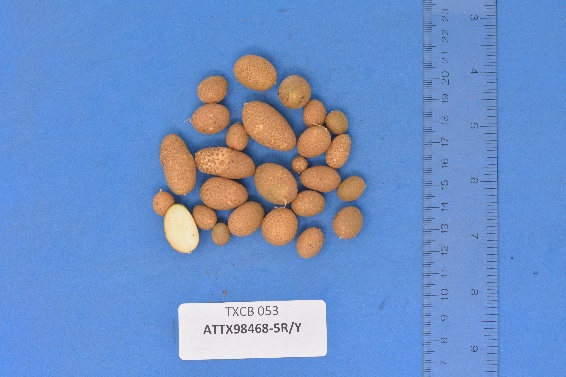

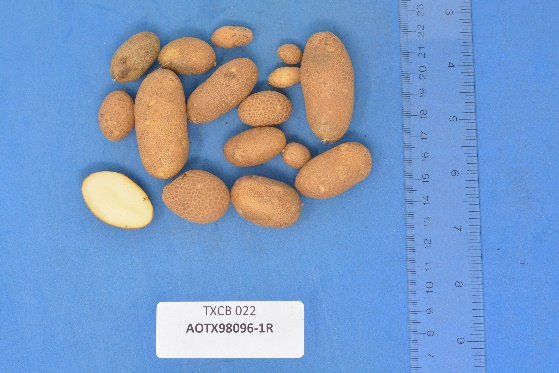
a. b.**

**
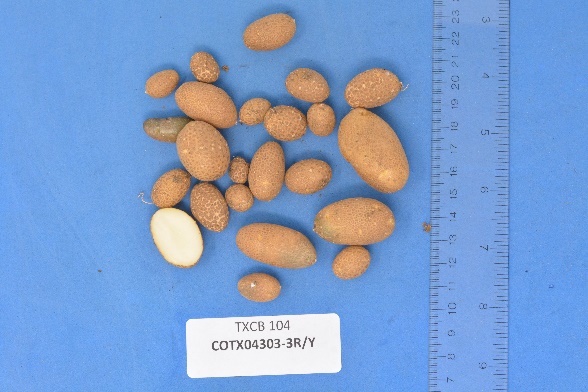

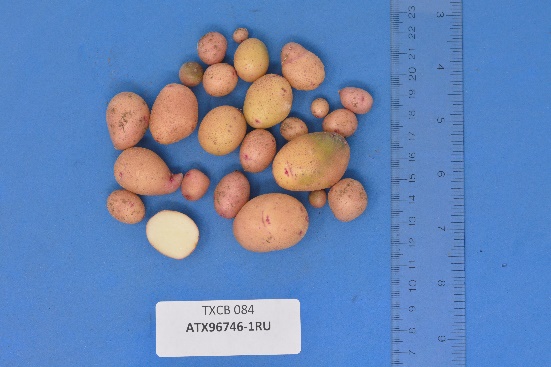
c. d.**

**
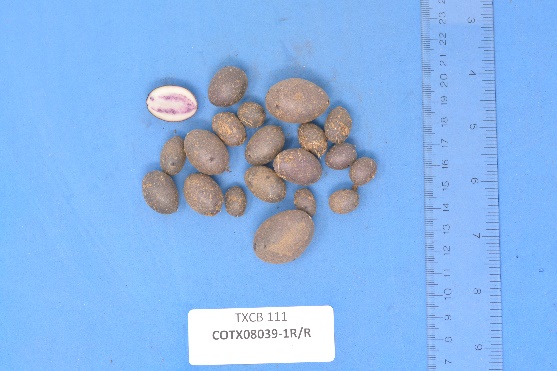
e.**

**Figure S5.** Illustration of skin and Flesh color verification from minitubers when the naming error was found from phylogenetic analysis. **a.** AOTX98096-1R correct name AOTX98096-1Ru **b.** ATTX98468-5R/Y correct name ATTX98468-5Ru/Y **c.** ATX96746-1Ru correct name ATTX96746-1R **d.** COTX04303-3R/Y correct name COTX04303-3Ru/Y **e.** COTX08039-1R/R correct name COTX08039-1P/P


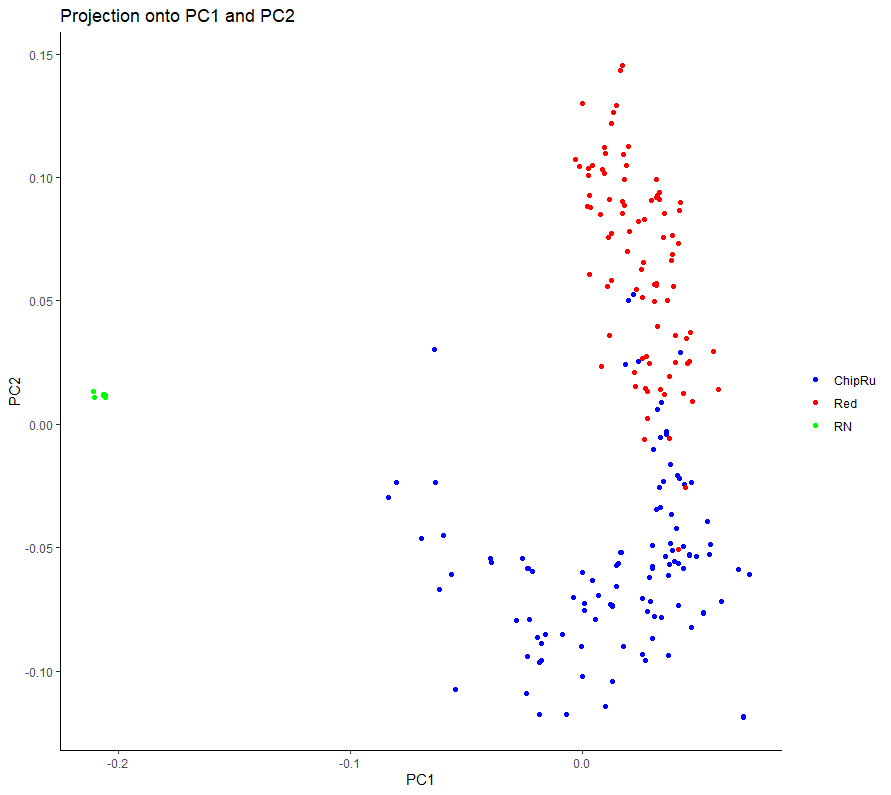


**Figure S6.** Score plot showing the population structure (first two principal components PC1 and PC2) for the observed data set produced with PCAdapt^72^ where dots correspond to individuals and color indicates sub-populations (ChipRu = Blue, Red = Red and RN = Green).


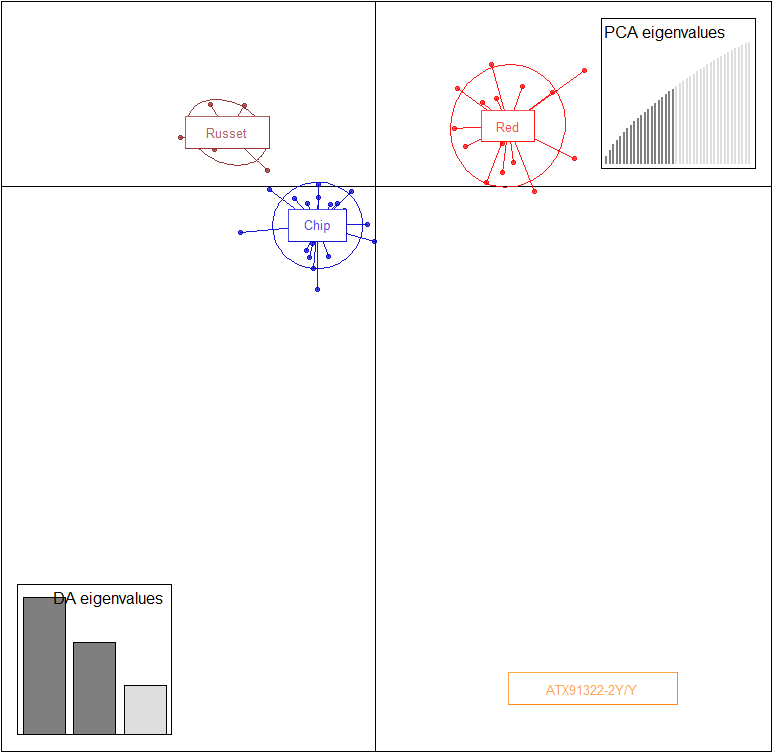


**Figure S7.** Discriminant analysis of principal components (DAPC) for 43 clones in the core set using *adegenet* R package^67^. The axes represent the first two linear discriminants (LD). Circles represent groups and dots represent individual clones. Numbers represent the different groups identified by DAPC analysis (Russet, Red and Purple and Chip and ATX91322-2Y/Y –-diploid-).


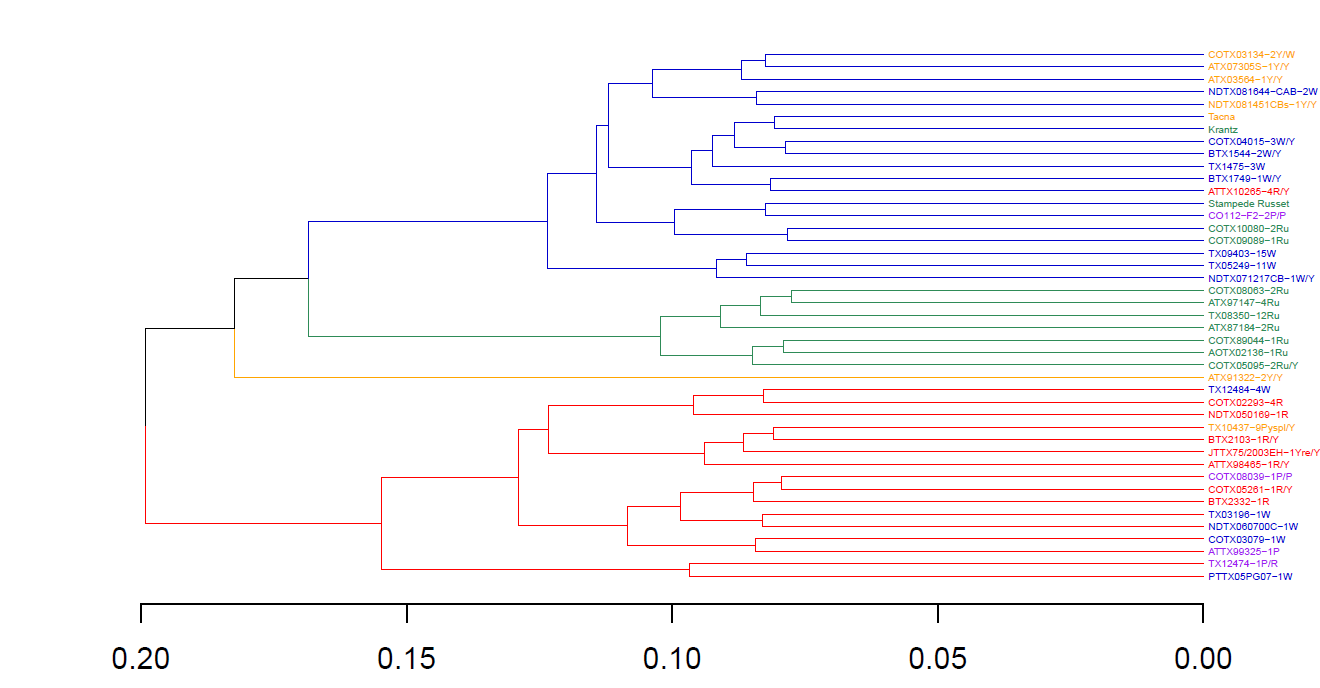


**Figure S8.** Dendrogram of the core set (43 genotypes) divided into clusters using Ape R package^70^. In the X-axis represents the Nei’s genetic distances between clones. In the Y-axis are represented the subpopulations by color in the core set. The color of the clones represents the market class (Red = red clones, Purple = purple clones, and Yellow = yellow clones; Green = Russet Clones, and; Blue = chipping clones)


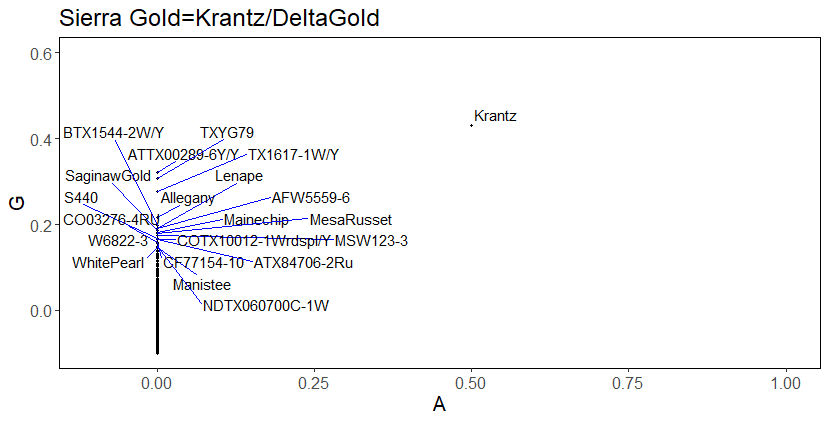


**Figure S9.** Population-wide comparison of genetic covariance calculated from markers with the additive relationship calculated from pedigree records, for clone Sierra Gold using R software^82^ with one parent (Krantz) genotyped.


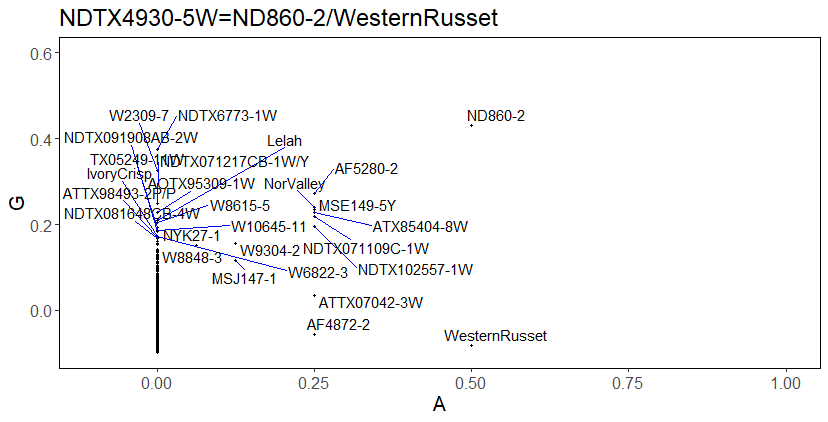


**Figure S10.** Population-wide comparison of genetic covariance calculated from markers with the additive relationship calculated from pedigree records, for clone NDTX4930-5W using R software^82^ in which one of the parent Western Russet seems to be erroneous.


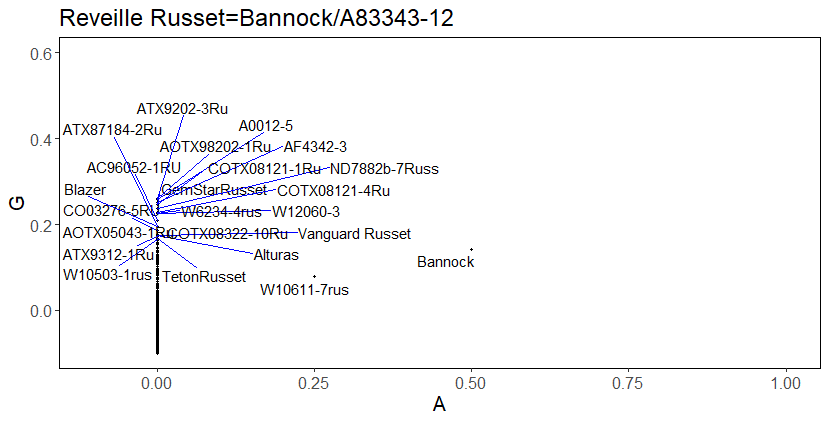


**Figure S11.** Population-wide comparison of genetic covariance calculated from markers with the additive relationship calculated from pedigree records, for clone Reveille Russet using R software^82^ in which one of the genotyped parent Bannock seems to be erroneous.
